# Combined Microscopy, Calorimetry and X-ray Scattering Study of Fluorinated Dimesogens

**DOI:** 10.1038/s41598-017-12799-1

**Published:** 2017-10-17

**Authors:** Richard J. Mandle, Stephen J. Cowling, John W. Goodby

**Affiliations:** Department of Chemistry, University of York, Heslington, YO10 5DD UK

## Abstract

The material FDO11DFCB3 (compound 2 in this work) remains the only example of a liquid-crystalline material to exhibit a phase transition from the heliconical twist-bend phase into a lamellar smectic A mesophase, additionally this material exhibits a previously unidentified mesophase. We have prepared and characterised several homologues of this compound, with each material subjected to an in-depth analysis by optical microscopy, calorimetry and small angle X-ray scattering studies. Despite FDO11DFCB3 being similar in chemical structure to the novel materials presented herein its liquid-crystalline behaviour is rather different, indicating an unexpected sensitivity of the twist-bend phase to molecular structure.

## Introduction

The discovery of the twist-bend phase (N_TB_) in liquid-crystalline dimers^1–21^ and later bent-cores^22^ and oligomers^23–26^ has given impetus to the study of mesomorphic materials of unusual molecular architecture. Several aspects of the N_TB_ phase have been reviewed recently^27–29^, and the consensus has been that the local structure of the phase is helical with a pitch length in the region of 10 nm^22,30,31^. This outcome, however, has been disputed^32,33^, and alternate models proposed^34^. Furthermore it has been clearly demonstrated that the incidence of the twist-bend phase is not especially sensitive to chemical composition^35,36^, with the only prerequisite being that a material is sufficiently bent – with the degree of bend having some control over the (relative) thermal stability of the phase^35^. When the twist-bend phase is chiral other ‘nematic-like’ mesophases have been reported whose structure is as-yet-unknown^8,37^, whereas a nematic-to-nematic transition has also been recently reported for a polar rod-like compound^38^.

**Figure 1:**
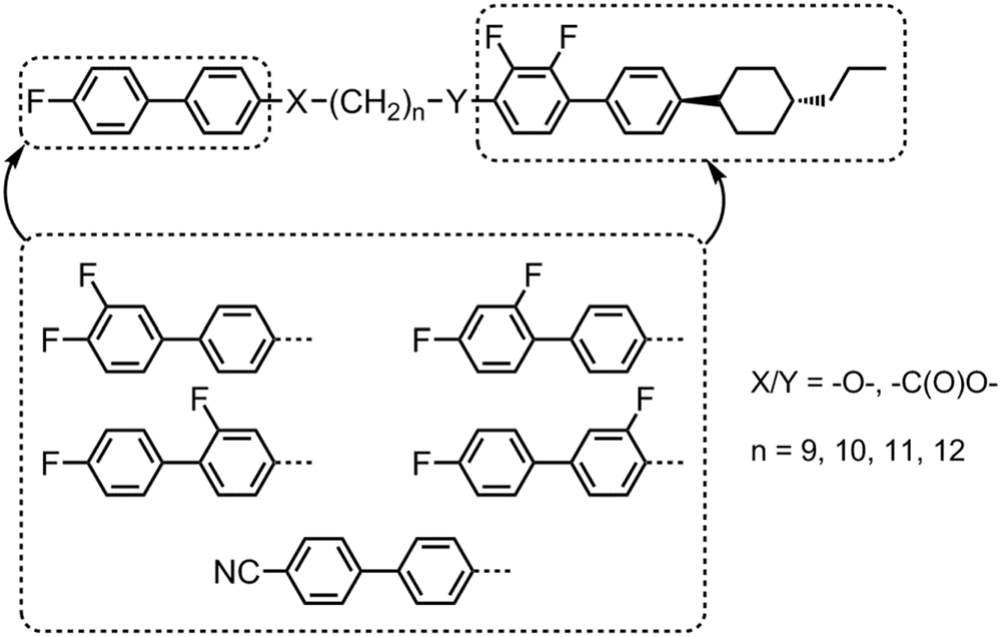
Some of the structural variations to compound **2** (Y = O, n = 11) that have been explored to date^2,39^.

Previously we reported the synthesis and characterisation of FDO11DFCB3 (compound **2** in this work) and this remains the only example of the phase sequence N-N_TB_-SmA that we are aware of, with the identifications of both the N, twist-bend and SmA phases confirmed by miscibility^39^. This phase sequence presents something of a unique testbed for theoretical treatments of the twist-bend phase; however compound **2** is far from ideal for study because the phases are both monotropic and exhibited only over a short temperature range. In a related study we demonstrated that the smectic A phase was suppressed when either mesogenic unit was replaced with a number of different groups (see Fig. 1), however in most cases the twist-bend phase was retained^2,39^. In this work we expand on our previous studies of **2**, and in the process preparing three new homologues with v terminal chain lengths, and presenting in depth studies of these materials by small-angle X-ray scattering (SAXS).

## Experimental

**Figure 2:**
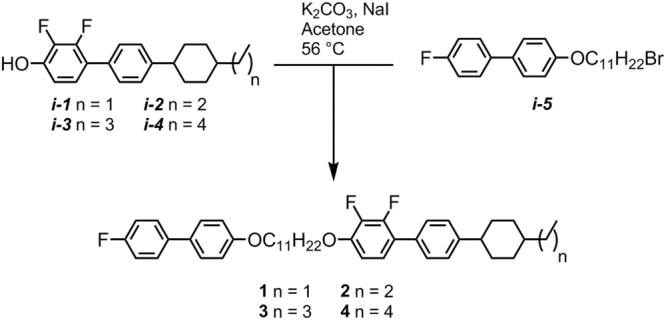
The synthetic route employed to compounds **1**–**4**.

The intermediate *trans* 4-(4-alkylcyclohexyl)-2′,3-difluoro-4′-hydroxybiphenyls (***i-1*** – ***i-4***) were available in house, other chemical intermediates were obtained from commercial suppliers. The intermediate ***i-5*** was prepared as described previously^39^. Williamson etherification of ***i-1*** – ***i-4*** with ***i-5*** afforded compounds **1–4**, as shown in Fig. 2. Full experimental details, including chemical characterisation and descriptions of instrumentation used, are available in the ESI to this article. Computational chemistry was performed at the B3LYP/6-31 G(d) level of DFT as implemented in Gaussian G09 revision e01^40^. Small angle X-ray diffraction was performed using a Bruker D8 Discover equipped with a temperature controlled, bored graphite rod furnace, custom built at the University of York. The radiation used was copper Kα (λ = 0.154056 nm) from a 1 μS microfocus source. Diffraction patterns were recorded on a 2048 × 2048 pixel Bruker VANTEC 500 area detector set at a distance of 121 mm from the sample, allowing simultaneous collection of small-angle and wide-angle scattering data. Samples were filled into 0.9 mm OD glass capillary tubes and aligned with a pair of 1 T magnets with the field perpendicular to the incident X-ray beam. Diffraction patterns were collected as a function of temperature (controlled to an accuracy of +/−0.1 °C). Two dimensional diffraction patterns were radially averaged (0.05° step size) to give one-dimensional profiles of scattered intensity as a function of two-theta, conversion into d-spacing (Å) or scattering vector (Q) is then trivial.

## Results

The liquid-crystalline behaviour of compounds **1–4** was studied by a combination of polarised optical microscopy (POM), differential scanning calorimetry (DSC) and small angle X-ray scattering (SAXS). Transition temperatures and associated enthalpies of transition were determined by DSC and are presented in Table 1. Phase assignments were made based on combinations of POM and SAXS studies as described in the text.

**Table 1:** Transition temperatures (°C) and associated enthalpies of transition [kJ mol^−1^] for compounds **1–4**, as determined by DSC at a heat/cool rate of 10 °C min^−1^.

No.	n	Cr		Sm_Y_		Sm_X_		SmA		N_TB_		N		Iso
1	1	•	76.1 [44.2]	—	—	(•	57.1 [0.5]	•	67.0) [0.3]	—	—	•	148.3 [1.9]	•
2	2	•	79.8 [47.4]	—	—	(•	58.5 [0.1]	•	66.7 [0.6]	•	68.8) [<0.1]	•	162.9 [2.4]	•
3	3	•	74.1 [42.3]	(•	55.4 [<0.1]	•	73.6) [0.5]	•	91.9 [0.4]	—	—	•	159.6 [1.8]	•
4	4	•	74.2 [43.3]	—	—	(•	61.5) [0.5]	•	80.4 [0.2]	—	—	•	158.6 [2.5]	•

Transitions in parenthesis are monotropic, *i.e*. they occur below the melting point of the sample. For chemical structures see Fig. 2.

As shown previously, **2** exhibits the phase sequence N-N_TB_-SmA with an additional and as yet unidentified smectic phase (Sm_X_)^39^. If the length of the terminal alkyl chain of the 4′-(4-alkylcyclohexyl)-2,3-difluorobiphenyl mesogenic unit (CDFB) is shortened (**1**) or increased (**3** and **4**) then the N_TB_ phase is not observed. All four materials exhibit N, SmA and unidentified Sm_X_ phases with compound **3** also exhibiting an additional as-yet unidentified Sm_Y_ phase. The Sm_X_-Sm_Y_ transition is first order and occurs with a vanishingly small associated enthalpy, suggesting the two phases are closely related in structure.

**Figure 3:**
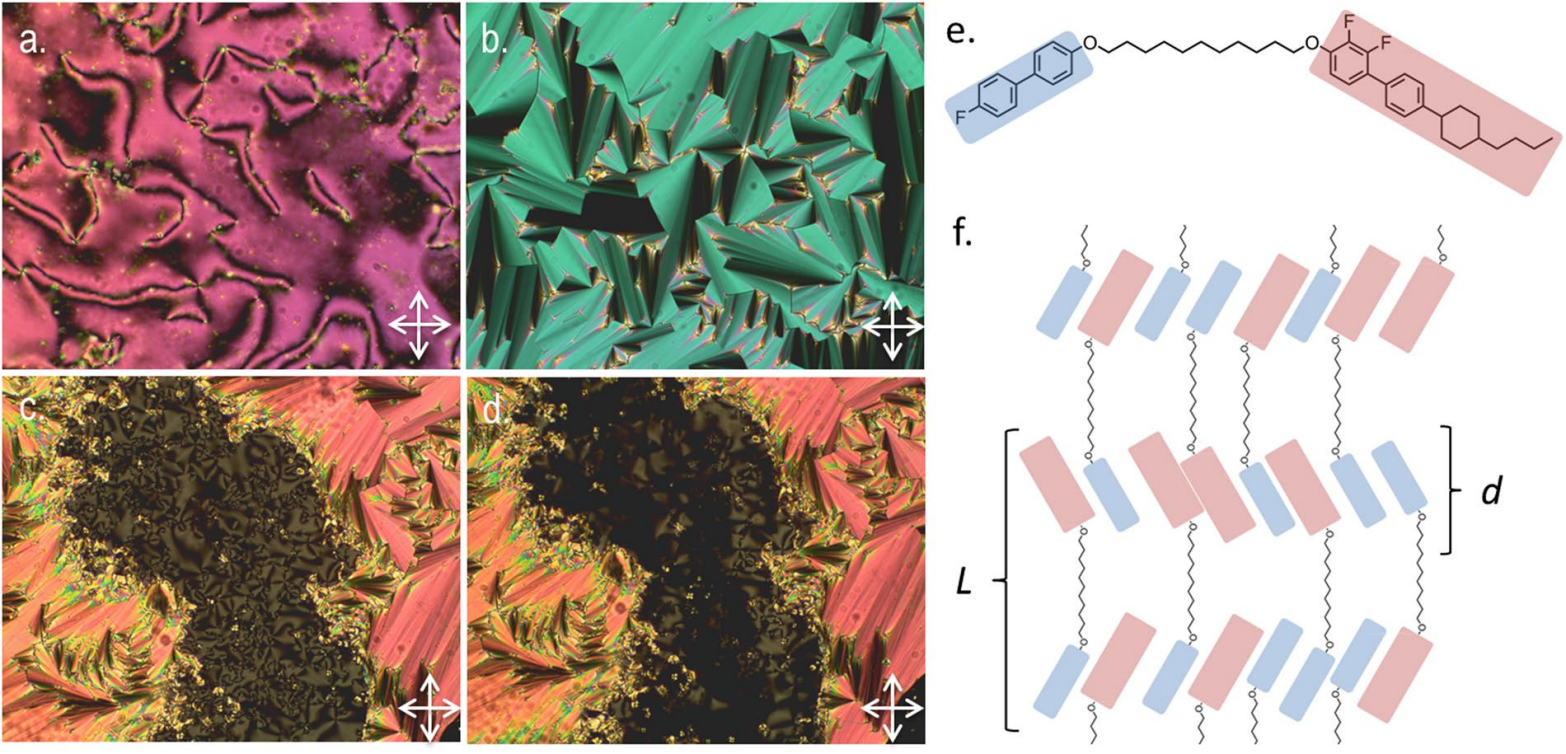
Photomicrographs (x100, crossed polarisers) of the *schlieren* texture of the nematic phase of **3** at 139.8 °C (**a**), the smectic A phase of **3** at 83.0 °C (**b**), the focal-conic and *schlieren* textures of the anticlinic smectic C phase (SmC_A_, denoted as Sm_X_ in Table 1) of **3** at 65.6 °C (**c**) and approximately the same region of the sample at the phase transition from the SmC_A_ phase to the Sm_Y_ phase at 59.9 °C (**d**). The molecular structure of compound **3** (**e**), mesogenic units are colour coded as used in the schematic depiction of the Smectic C_A_ phase shown in (**f**). The molecular length (L) and smectic layer spacing (**d**) are indicated.

The identification of the nematic and smectic A phases of **1–4** was trivial based on their *schlieren* and focal-conic defect textures respectively, whereas the identification of the twist-bend phase for compound **2** was demonstrated previously. The Sm_X_ phase of **3** has a higher thermal stability than for **2** (or for that matter **1** or **4**), with this material also exhibiting an additional unknown ‘Sm_Y_’ mesophase. The Sm_X_ phase was identified as a smectic C analogue based on the appearance of a *schlieren* texture in optically extinct regions of the SmA phase (Fig. 3c). As the *schlieren* texture features both 2- and 4- brush defects it can be identified as having anticlinic layer organisation, which is to say that the tilt direction alternates from layer-to-layer. A number of dimeric liquid crystals having odd spacer parity have been reported to exhibit anticlinic smectic C phases (SmC_A_)^3,5,19,36^. Further cooling of the SmC_A_ phase into the ‘Sm_Y_’ phase (Fig. 3d), the *schlieren* texture is retained and is made up of both 2- and 4- brush defects, confirming the tilted, anticlinic nature of this phase. This indicates that the ‘Sm_Y_’ phase is a tilted smectic, and is either SmF_A_ of SmI_A_ (there are no other anticlinic phases that exhibit a schlieren texture that we are aware of) or it is a new mesophase.

**Figure 4:**
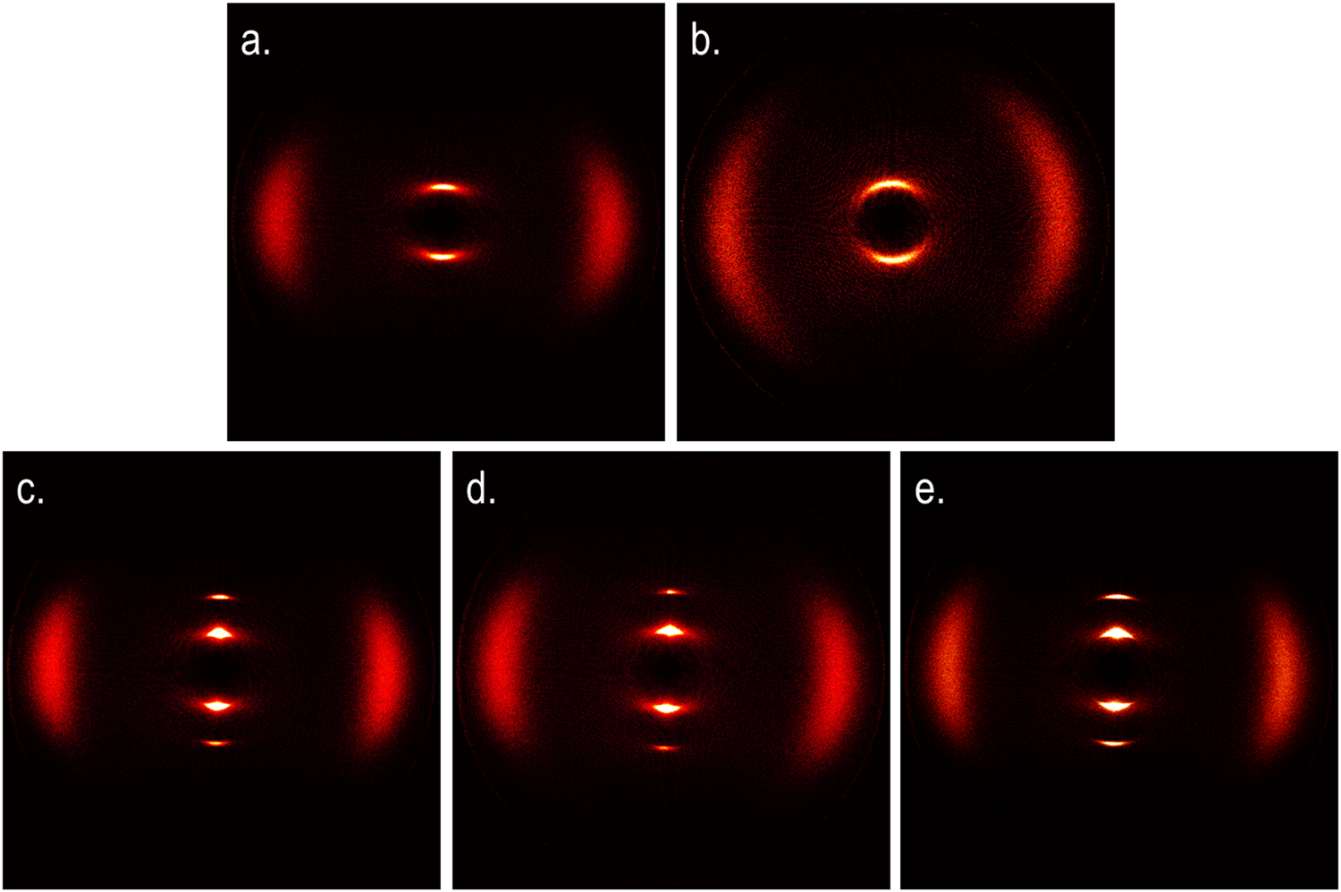
Two dimensional small angle X-ray scattering patterns obtained for magnetically aligned samples: (**a**) the nematic phase of **4** at 85 °C; (**b**) the twist-bend phase of **2** at 67 °C; (**c**) the SmA_1_ phase of **3** at 75 °C; (**d**) the SmC_A_ phase of **1** at 52 °C; (**e**) the ‘Sm_Y_’ phase of **3** at 50 °C.

We now turn to the study of compounds **1–4** by small angle X-ray scattering. The aim of this study was to confirm (or refute) the assignment of the smectic mesophases exhibited by **1–4**, and determine the subtype of the smectic A phase. Representative 2D SAXS patterns are given in Fig. 4. Each two dimensional pattern was radially averaged (0.05° step size) to give scattered intensity versus 2θ; the data was fitted using 3-term Gaussian functions to obtain peak positions and correlation lengths both parallel and perpendicular to the director. The 2D SAXS patterns obtained for each mesophase are consistent with the phase identifications made by microscopy. For the nematic (Fig. 4a) and twist-bend (Fig. 4b) phases there is only diffuse scattering at small angles due to the lack of positional order of the molecules. For the smectic phases (Fig. 4c and d) the lamellar structure is revealed by sharp Bragg scattering, whilst diffuse scattering at wide-angles indicates a lack of in-plane organisation, and thereby refutes the possibility that these mesophases are higher ordered smectic soft crystals.

Despite cooling compound **3** below the temperature at which the SmC_A_-‘Sm_Y_’ transition occurs we did not observe any change in the 2D SAXS pattern (Fig. 4e), the peak positions or the correlation lengths. As discussed previously, the combination of focal-conic and *schlieren* textures demonstrate that the mesophase is a tilted smectic. Although the *schlieren* texture changes markedly at the phase transition (see Fig. 3c and d) both 2- and 4- brush defects appear to remain in the lower temperature phase, indicating an anticlinic organisation. However, based on SAXS data the possibility of the Sm_Y_ mesophase being tilted smectic phase with long range orientational order (SmI, SmF) can be ruled out due to the lack of sharp scattering at wide angles, which would be indicative of the long in-plane correlation lengths present for such a phase. Instead the wide-angle scattering is diffuse and therefore indicative of a lack of in-plane ordering. However, this does not rule out the possibility of a SmI or SmF phase with long range bond-orientational order, which would show a similarly diffuse wide-angle scattering pattern. The vanishingly small enthalpy associated with the transition – coupled with the SAXS patterns – means that we feel the most likely explanation is that this is a transition between two subtypes of SmC phase, the higher temperature phase being SmC_A_. Whilst we are not able to definitively identify the lower temperature phase presently, transitions between different subtypes of the SmC phase (such as modulated smectic C phase ($Sm\tilde{C}$), smectic C antiphase (SmC_anti_)) are known. More speculatively, this phase could be an example of a smectic twist-bend phase (Sm_TB_?) although we concede that such a state of matter is hypothetical presently.

Rather than use the d-spacing of the small angle scattering peak we convert to a d/l ratio (*i.e*. the ratio between the d-spacing of the small angle scattering peak and the molecular length), allowing us to compare materials of differing length. Molecular lengths were calculated on isolated molecules in their all *trans* geometries at the B3LYP/6-31 G(d) level of DFT (**1**: 39.9 Å, **2**: 40.8 Å, **3**: 41.8 Å, **4**: 42.8 Å). Clearly this neglects the flexibility that is inherent to a dimer with an undecamethylenedioxy spacer, however for the present use in the analysis of SAXS data it is sufficient and we will return to this point shortly.

**Figure 5:**
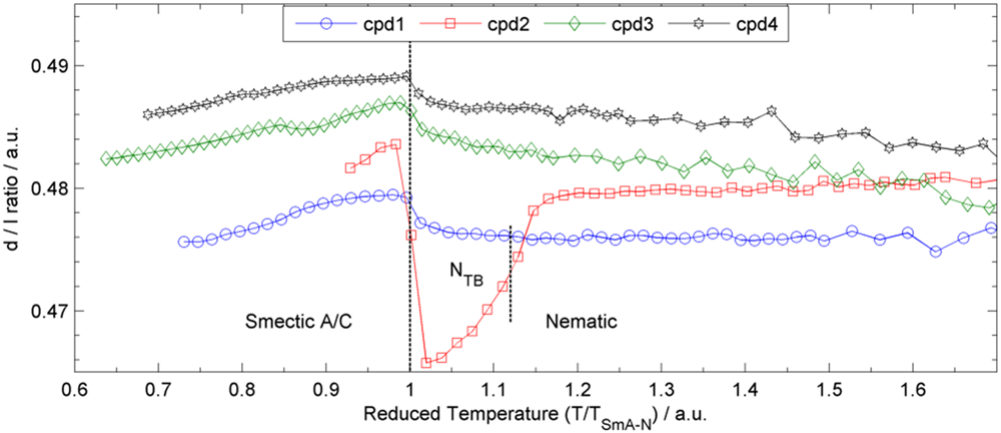
Plot of the d/l ration of the major small angle scattering peak as a function of reduced temperature (T/T_SmA-N_, or for compound **2** T/T_SmA-NTB_) for compounds **1–4**. The dashed line corresponds to the SmA-N phase transition at a reduced temperature of 1 (or SmA-N_TB_ in the case of **2**). An additional dashed line indicates the N_TB_-N transition temperature for compound **2**. In the case of **2** the material crystallised during SAXS study prior to the onset of the SmC phase. Assuming the reduction in d-spacing across the N_TB_ phase – relative to the d-spacing in the nematic - is a consequence of the tilting of molecules with respect to the helical axis then the conical angle can be estimated from equation 1.

A plot of the d/l ratio of **1–4** as a function of reduced temperature is given in Fig. 5. The data is shows noise in the nematic phase (reduced temperature of >1) due to the diffuse nature of the small angle scattering. Compound **2** possesses a twist-bend phase in addition to the mesophases exhibited by compounds **1**, **3** and **4**, and it is unsurprising that when studied by SAXS some of its behaviour is unique. Firstly the twist-bend phase range during SAXS experiments appears to be significantly larger than determined by POM/DSC: the onset temperature of the N_TB_ phase (as judged by the minor change in the SAXS pattern, see Fig. 4a and b) is at ~74 °C (rather than 68.8 °C by DSC), however the SmA-N_TB_ transition temperature remains unchanged. The temperature of the graphite-rod furnace is known to be stable to +/−0.1 °C and so this can be excluded. We also exclude the confined geometry of the capillary as the internal diameter used is ~0.85 mm and is therefore far larger than the gap between slide and coverslip during POM. It is known that for flexible dimers experience anomalous increases in transition temperatures when in a very large magnetic field (~22 T)^41^. However it would be surprising for a near 6 °C increase in T_NTB-N_ to be attributable to the rather weak aligning magnetic field used here (~1 T). Secondly, as shown by the dashed line separating the nematic and twist-bend mesophases, there is a notable decrease in the d/l ratio across not only the entire N_TB_ phase range but also in the nematic phase close to the transition. This reduction is a consequence of the molecules reorganising tilting away from the helical axis upon entering the heliconical twist-bend phase. Using the average d-spacing in the nematic phase range, we calculate that the conical angle reaches a maximum value of ~14° immediately prior to the onset of the SmA phase. It appears that this tilt away from the director begins in the nematic phase, suggesting some pretransitional change maybe taking place close to the N_TB_-N transition. Following the N_TB_-SmA phase transition in compound **2**, the diffuse small angle scattering peak is superseded by a sharp Bragg peak, and there is a significant increase in the d/l ratio, however the material crystallises prior to entering the SmC phase.

1 $$\varphi =a\,\cos \,\frac{{d}_{{N}_{TB}}}{{d}_{nematic}}$$

Turning now to the other materials, both the smectic A and smectic C mesophases of **1**, **3** and **4** are intercalated, with layer spacings of less than half a molecular length. The measured d/l ratios show a small dependence on the length of the terminal chain, with **4** having the largest layer spacing and **1** having the smallest. Only one major peak for layer spacing is observed in both smectic phases, and this strongly suggests there is no segregation of mesogenic units. The layer spacing in both the SmA and SmC phases is practically temperature independent (differing by only ~0.2 Å over the entire temperature range of both phases in **3**). SAXS data indicates that both smectic phases are intercalated monolayer phases, however in the case of the smectic C phase polarised optical microscopy indicates that the phase has anticlinic rather than synclinic layer organisation.

**Figure 6:**
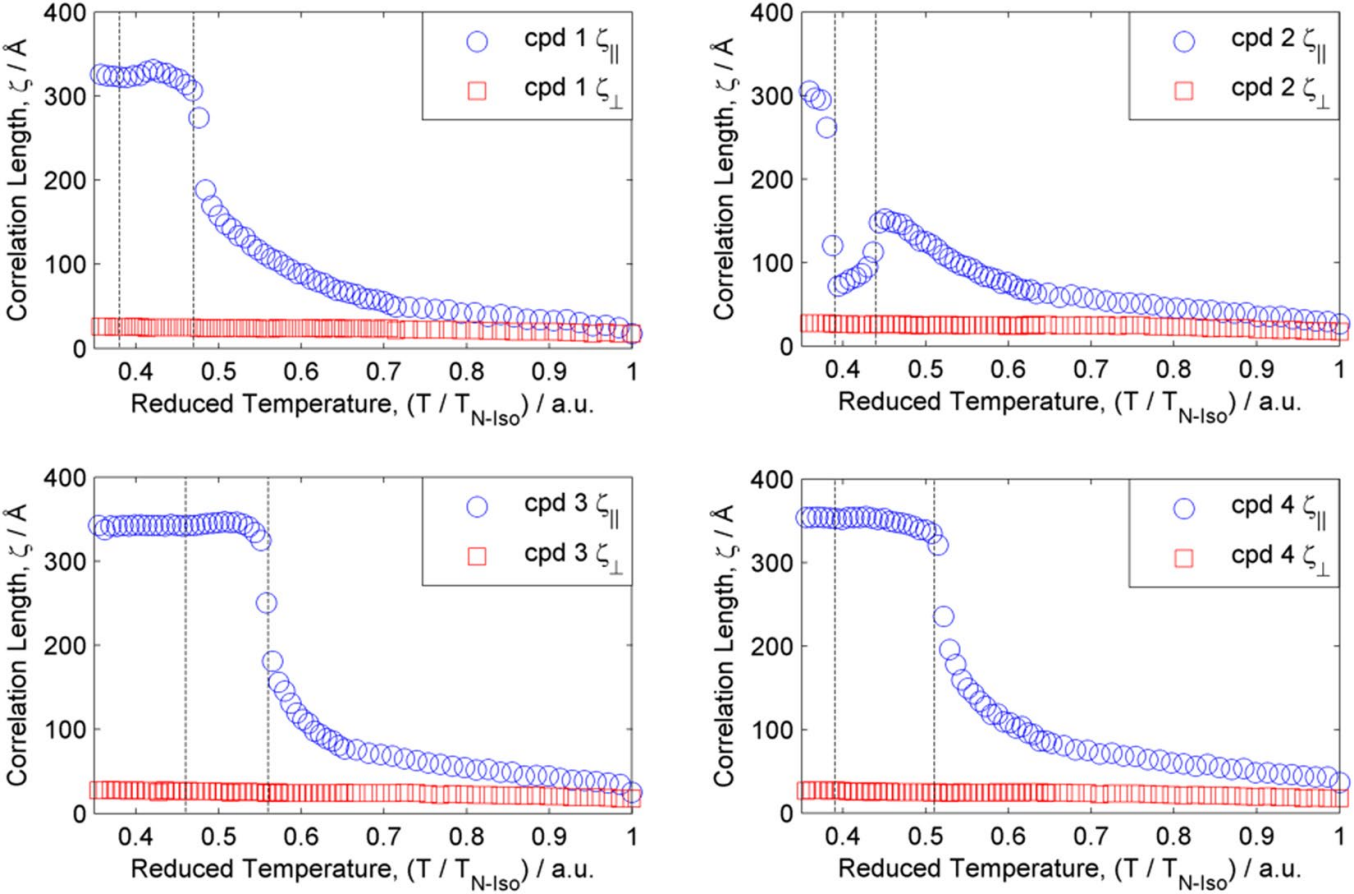
Plots of the correlation length parallel to the director (*i.e*. out of plane, ζ_||_) and perpendicular to the director (*i.e*. in plane, ζ_⊥_). Correlation lengths parallel to the director were determined by fitting small angle scattering data (2θ 2.25–8) with a Gaussian fit, from the full-width at half maximum of the fit we obtain the correlation length. Repeating this process on wide-angle scattering data affords the correlation length perpendicular to the director. The R^2^ value for each fit was typically >0.99, except in the isotropic liquid where it was lower due to the reduced scattered intensity. Dashed lines correspond to the location of phase transitions, as judged by the 2D SAXS patterns. Representative fit data used to extract correlation lengths is given in the ESI (Figure SI-21).

Determination of the full width half maximum of the major small- and wide- angle scattering peaks affords the correlation length parallel and perpendicular to the director. These were obtained for compounds **1–4** and are presented in Fig. 6. We observe that **1**, **3** and **4** follow broadly the same trend over the entire temperature range, with the correlation length within the plane exhibiting only a marginal increase (being almost temperature independent at each phase transition). The out-of-plane correlation length exhibits a continuous build up throughout the nematic phase range, but is effectively temperature independent in the smectic A and C mesophases. Compound **2** exhibits a twist-bend phase intermediate between the nematic and smectic A phases and consequently behavers somewhat differently to the other members of this homologous series. Throughout the nematic phase there is a steady build up in the correlation length parallel to the director, however upon entering the twist-bend nematic phase this decreases rapidly from ~150 Å at the N-N_TB_ phase transition to just ~70 Å at the N_TB_-SmA phase transition. Upon entering the smectic A_1_ phase there is a marked increase in the out-of-plane correlation length (which saturates at similar values to those obtained for **1**, **3** and **4**). For all four compounds the in-plane correlation length remains small throughout the entire liquid-crystalline temperature range; this is clearly expected for the nematic and twist-bend phases, but in the smectic A and C phases this observation confirms the lack of positional organisation and refutes the possibility of higher ordered smectic phases being present.

**Figure 7:**
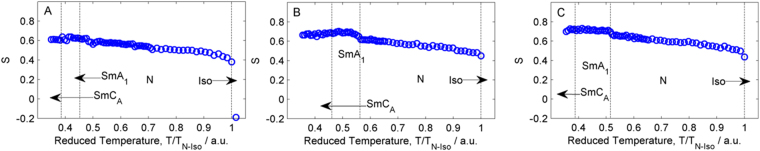
Plots of the order parameter (S) as a function of reduced temperature (T/T_N-Iso_) obtained as described in the text for compounds **1** (**a**), **3** (**b**) and **4** (**c**).

Using the method of Davidson *et al*.^42^ we obtained values of the orientational order parameter (S) for compounds **1, 3** and **4** as a function of reduced temperature across the whole phase range (Fig. 7). We could not obtain an order parameter for **2** due to poor quality of alignment of the sample. Compounds **1**, **3** and **4** behave similarly; there is a steady increase in the value of S with reduced temperature, saturating at ~0.6 prior to the N-SmA transition; this value increases to ~0.7 (0.64 in **1**) upon entering the SmA phase, before decreasing marginally into the SmC_A_ phase.

**Figure 8:**
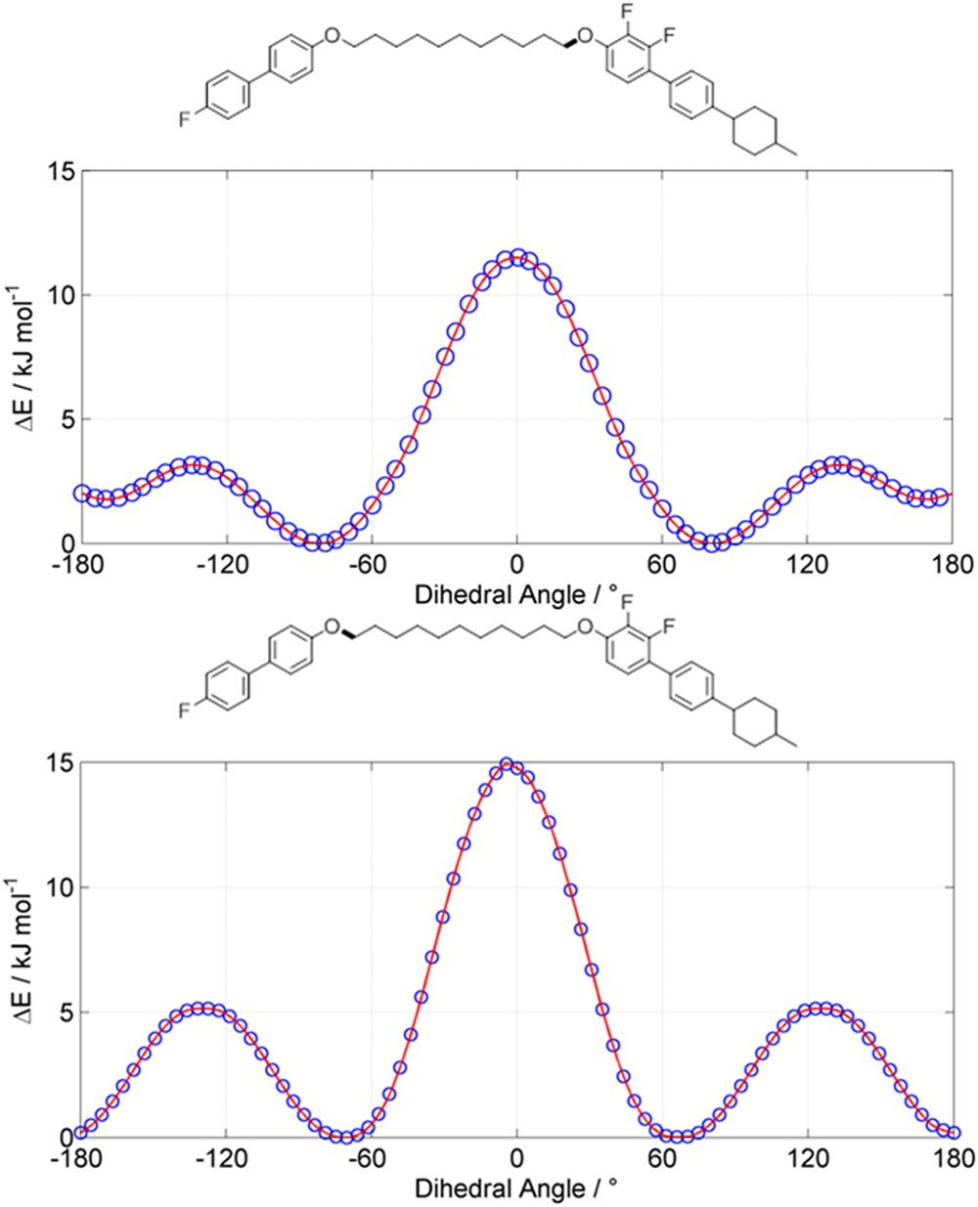
Plots of energy relative to the lowest energy conformer (ΔE, kJ mol^−1^) versus the dihedral angle for indicated torsions of a methyl-terminated analogue of compounds **1–4**, as determined with fully relaxed scans (71 × 5° steps) with the AM1 semi empirical method as implemented in Gaussian G09 e01^40^. Plots for other torsional angles are given in the SI to this article.

As noted in the introduction it is perhaps surprising that compounds **1–4** exhibit smectic A phases given that they have a gross bent shape. As far as is known, all current examples of twist-bend to smectic phase transitions the lamellar phase is tilted^5,19,36^, and this observation is easily rationalised given the anticipated bent shape of these materials. Efforts to obtain single crystals of **1–4** suitable for structure determination by XRD were unsuccessful. We therefore opted to explore the conformational landscape of **1–4**; replacement of the terminal chain with a methyl group reduces the complexity of the systems, however even using a simple *trans/*+ *gauche/-gauche* approximation the spacer alone has something like3^12^ conformers. We therefore elected to study each individual torsional angle present in the spacer employing the AM1 semi empirical method using fully relaxed scans in 71 × 5° steps *via* the MODREDUNDANT keyword in Gaussian G09 e01^40^. From these the lowest energy conformation was identified, allowing us to plot ΔE versus the resulting dihedral angle. For the ‘first’ and ‘last’ torsions (see Fig. 8) the *trans* form was higher in energy than the *gauche* by 1.7 and 0.2 kJ mol^−1^ respectively, however these serve to reduce the bend angle (*i.e*. more bent) rather than to straighten the molecule out. For all torsions, with the exception of the ‘first’ and ‘last’ (*i.e*. adjacent to the mesogenic units) the all *trans* conformation was the energy minima, plots are given in the ESI to this article (Figures SI18, SI19 and SI20). Given how close in energy many of the *trans* and *gauche* −/+ states are many conformers are likely to be significantly populated, however crucially these calculations show that **1–4** are most likely to be significantly bent rather than linear. The ^1^H NMR of **1–4** features many overlapping peaks (see ESI) which unfortunately precludes using selective 1D^1^H NOESY to obtain internuclear distances to support calculated geometries

Given that the materials exhibit smectic A mesophases we performed preliminary studies into their potential use as hosts in smectic A based scattering devices. A mixture of compound **3** – selected because of its relatively wide SmA phase range – doped with 0.5 wt% hexadecyltrimethylammonium perchlorate was prepared and the sample filled into a glass cell with ITO electrodes (spacing ≈ 50 μm, cells supplied by Halation). The behaviour in the nematic phase was unremarkable, with a Fréedericksz transition observed at low voltage and frequency. At higher applied voltages and frequencies a rich array of electrohydrodynamic instabilities (Williams domains) were observed. Similar behaviour was observed previously for compound **2** and a detailed description of this is given previously^39^. Following cooling into the smectic A phase (~2 °C below the N-SmA transition) we applied a voltage of 110 V across the cell at low and high frequencies (1 Hz and 1 kHz respectively). For a typical low molecular weight SmA material (such as 8CB) a low frequency applied field would transform the active area of the cell into a scattering state, the subsequent application of a high frequency then returns this to a clear state^43–46^. In the case of compound **3** however, we did not observe any switching processes. It may be that as the smectic A phase (and indeed the SmC phases) are intercalated the layers are significantly harder to disrupt *via* application of external electric fields. This means that dimers and dimesogens that have intercalated mesophase structures are likely to be unsuitable for use in display devices utilising smectic phases.

## Conclusions

Compound **2** remains the only known example of a material exhibiting nematic, twist-bend and smectic A mesophases. In previous work we demonstrated that variations to the molecular structure of compound **2** typically retained the twist-bend phase but the smectic A phase was eliminated. In this present work we present several homologues of **2** in which the terminal chain length has been varied, and we find that for these materials the SmA phase is retained at the expense of the twist-bend phase. This has, however, allowed us to determine that the lower temperature ‘X’ phase of **2** (and its homologues) is an anticlinic C phase. Compound **3** also exhibits an additional smectic phase, whose structure is unknown, however evidence from POM, DSC and SAXS points to a SmC-type mesophase – this material therefore may exhibit an uncharted SmC – SmC transition.

In a detailed X-ray scattering study of compounds **1–4** we observe that the behaviour of **1**, **3** and **4** is largely the same; the smectic layer spacing indicates intercalated layer structure whereas the correlation length out-of-plane (*i.e*. parallel to the director) shows a steady build up throughout the nematic phase before reaching values of ~300 Å in the smectic A phase. Compound **2** displays somewhat different behaviour; the correlation length increases throughout the nematic phase range to a maximum of 150 Å prior to the N-N_TB_ transition, across the entire N_TB_ phase range the out-of-plane correlation length decreases before reaching a value of ~300 Å in the smectic A phase. Additionally the d-spacing value of the small angle scattering peak in the nematic phase decreases across the twist-bend phase range, and this is probably understood as a consequence of the molecules (and the local director) tilting relative to the helical axis. For these compounds **1**, **3** and **4** the high quality alignment obtained during SAXS study allowed us to obtain orientational order parameters; in the nematic phase the values are consistent with those reported previously for bent dimers^47^, whereas the values in the smectic A and C phases show a sharp increase at the phase transition. Finally, we note that intercalated smectic phases as found for compounds **1** to **4** (and related dimesogens) do not respond appropriately to applied electric fields, and therefore are of minimal use in display devices with the exception of use in formulating mixtures.

## Electronic supplementary material

supplementary info
