# Supplementary material for: Combined Microscopy, Calorimetry and X-ray Scattering Study of Fluorinated Dimesogens
Source: Sci Rep. 2017 Oct 17;7:13323. doi: 10.1038/s41598-017-12799-1 (PMC5645320; doi:10.1038/s41598-017-12799-1)
Supplement: Supplementary file 1 — supplementary info [file 41598_2017_12799_MOESM1_ESM.pdf]

Supplementary Information for

Combined Microscopy, Calorimetry and X-ray Scattering Study of Fluorinated Dimesogens

Richard J. Mandle *, Stephen J. Cowling and John W. Goodby

Department of Chemistry, University of York, YO10 5DD, UK

* Richard.mandle@york.ac.uk

1 Synthesis:

1.2 General Methods.

1.2.1. Chemical Characterisation.

All chemicals were purchased from commercial suppliers and used as received, without further purification with the exception of solvents which were dried *via* percolation through active alumina and deoxygenated with Q5 copper catalyst prior to use. Intermediate compounds ***i-1***, ***i-2***, ***i-3*** and ***i-4*** were available in house, while ***i-5*** and compound **2** were prepared as described previously. [1,2] Reactions were monitored by thin layer chromatography (TLC) using an appropriate solvent system. Silica coated aluminium TLC plates used were purchased from Merck (Kieselgel 60 F-254) and visualised using either UV light (254 nm and 365 nm), or by oxidation with either iodine or aqueous potassium permanganate solution. Yields refer to chromatographically (HPLC) and spectroscopically (^{1}H NMR, ^{13}C{^{1}H} NMR and ^{19}F NMR) homogenous material.

NMR spectra were recorded on a JEOL ECS spectrometer operating at 400 MHz (^{1}H), 100.5 MHz (^{13}C{^{1}H}) or 376.4 MHz (^{19}F) as solutions in $CDCl_3$, unless stated otherwise. FT-IR spectroscopy was performed using a Shimadzu IR Prestige-21 with temperature controlled Specac Golden Gate diamond ATR IR insert. Liquid injection field desorption ionisation (LIFDI) mass spectra were performed on a Walters Micromass GCT premier orthogonal TOF instrument, atmospheric pressure chemical ionisation (APCI) mass spectra were acquired on a Bruker solariX XR FTMS instrument equipped with a 9.4 T magnet. We are grateful to Mr. Karl Heaton of the University of York for acquiring mass spectra.

Elemental analysis data (CHN) were obtained using an Exeter Analytical Inc. CE-440 Analyser and Sartorius S2 analytical balance. Calibration was performed against acetanilide standards and checked by the use of S-benzyl thiouronium chloride as internal standard (analytical grade, Exeter Analytical). We extend our gratitude to Dr. Graeme Mcallister of the University of York for performing elemental analysis.

High-performance liquid chromatography (HPLC) was performed on a Shimadzu Prominence modular HPLC system comprising a LC-20A quaternary solvent pump, a DGU-20A$_5$ degasser, a SIL-20A autosampler, a CBM-20A communication bus, a CTO-20A column oven, and a SPO-20A dual wavelength UV-vis detector operating at

230/255 nm. The column used was an Alltech C18 bonded reverse-phase silica column with a 5 μm pore size, an internal diameter of 10 mm and a length of 250 mm. The mobile phase used was neat acetonitrile that was sparged with nitrogen before use.

Computational chemistry was performed using the using Gaussian G09 revision e01 on the York Advanced Research Computing Cluster (YARCC) as described in the text. [3]

1.2.2. Characterisation of Mesomorphic Behaviour

Polarised optical microscopy was performed on a Zeiss Axioskop 40Pol microscope using a Mettler FP82HT hotstage controlled by a Mettler FP90 central processor. Photomicrographs were captured *via* an InfinityX-21 MP digital camera mounted atop the microscope. Differential scanning calorimetry was performed on a Mettler DSC822^e calibrated before use against indium and zinc standards under an atmosphere of dry nitrogen. In all cases a heat/cool rate of 10 °C min^{-1} was used, with presented values being the average of three runs.

Small angle X-ray diffraction was performed using a Bruker D8 Discover equipped with a temperature controlled, bored graphite rod furnace, custom built at the University of York. The radiation used was copper Kα (λ = 0.154 nm) from a 1 μS microfocus source. Diffraction patterns were recorded on a 2048x2048 pixel Bruker VANTEC 500 area detector at a distance of 121 mm from the sample, allowing simultaneous observation of both the wide angle (2θ = 15 – 25) and small angle (2θ = 3 – 15) scattering. Samples were filled into 1mm capillary tubes and aligned with a pair of 1 T magnets, the field strength at the sample position being approximately 0.6 T. Diffraction patterns were collected as a function of temperature and the data processed using Matlab. Raw data are available upon request from the University of York data catalogue.

1.3. General Reaction Procedure

A suspension of ***i-1*** – ***i-4*** (0.6 mmol), ***i-5*** (200 mg, 0.63 mmol), potassium carbonate (138 mg, 1 mmol) and sodium iodide (1 g) in acetone (30 ml) was heated under reflux with stirring for 48 h, after which time TLC analysis showed complete consumption of ***i-5***. The reaction was cooled, diluted with dichloromethane, filtered to remove insoluble matter and concentrated *in vacuo*. The crude material was purified by flash chromatography with 2:1 hexanes/DCM as the eluent, and the chromatographed material concentrated *in vacuo* to afford a white solid. This solid was redisolved in the minimum quantity of DCM and passed through a 0.2 μm nylon filter to remove particulates. The DCM solution was then passed through a plug of neutral alumina, eluting with a small quantity of dichloromethane. Hexane was carefully added to this solution until the onset of turbidity, at which point the sample was gently heated to dissolve any precipitated material. The now homogenous solution was cooled to -20 °C to induce precipitation, affording the title compounds as fine white solids.

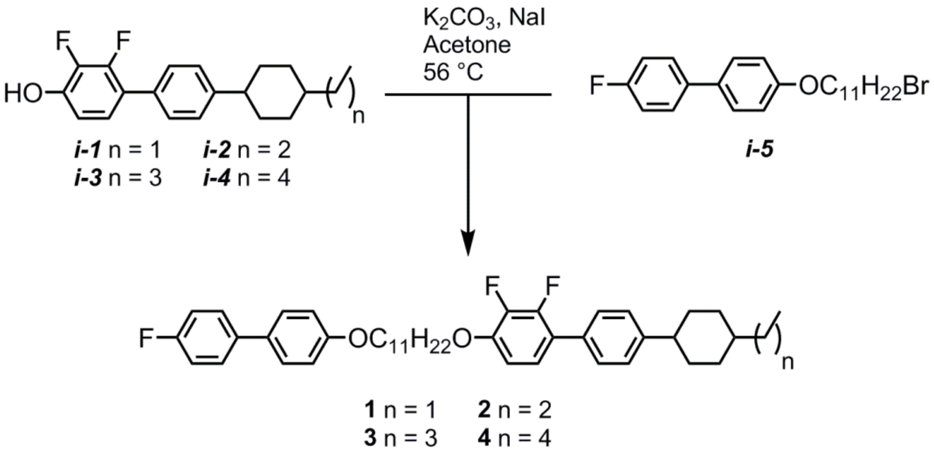

Scheme 1

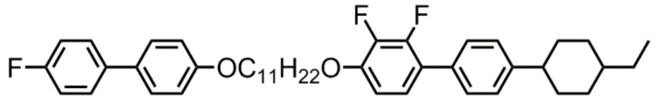

2,3-Difluoro-4-((11-((4'-fluoro-[1,1'-biphenyl]-4-yl)oxy)undecyl)oxy)-4'-(4-ethylcyclohexyl)-1,1'-biphenyl (1)

Quantities used:	***i-1*** (252 mg, 0.6 mmol), ***i-5*** (200 mg, 0.63 mmol), potassium carbonate (138 mg, 1 mmol) and sodium iodide (1 g) in acetone (30 ml). The general experimental procedure afforded the title compound as a white solid.
Yield:	240 mg (61%)
^{1}H NMR (400 MHz, $CDCl_3$):	0.91 (3H, t, *J* = 7.3 Hz, Cy-CH_2-CH_3), 1.05 (2H, dquartet, *J* = 4.1 Hz, *J* = 13.7 Hz, Cy*H*), 1.16 – 1.52 (20H, m, Cy-CH_2-CH_3 + ArO-CH_2-CH_2-(CH_2)$_7$-CH_2-CH_2-OAr' + CyH_4), 1.73 – 1.96 (8H, m, ArO-CH_2-CH_2-(CH_2)$_7$-CH_2-CH_2OAr' + ChH_2), 2.49 (1H, tt, *J* = 3.2 Hz, *J* = 12.4 Hz, Cy*H*), 3.98 (2H, t, *J* = 7.3 Hz, ArO-CH_2-CH_2), 4.05 (2H, t, *J* = 7.3 Hz, ArO-CH_2-CH_2), 6.76 (1H, ddd, *J* = 1.8 Hz, *J* = 7.8 Hz, *J* = 8.7 Hz, Ar*H*), 6.94 (2H, ddd, *J* = 2.3 Hz, *J* = 2.8 Hz, *J* = 9.2 Hz, Ar*H*), 7.03 – 7.11 (3H, m, Ar*H*), 7.25 – 7.28 (2H, m [obscured by $CHCl_3$ peak], Ar*H),* 7.38 – 7.51 (6H, m, Ar*H*)
^{13}C{^{1}H} NMR (100.5 MHz, $CDCl_3$):	11.52, 25.85, 26.02, 29.13, 29.26, 29.29, 29.35, 29.46, 29.48, 29.58, 29.97, 33.13, 34.23, 39.04, 44.13, 68.06, 69.82, 109.46 (d, $J_{C\text{-}F}$ = 2.9 Hz), 114.77, 115.36, 115.59, 122.96 (d, $J_{C\text{-}F}$ = 11.5 Hz), 123.45 (t, $J_{C\text{-}F}$ = 4.8 Hz), 127.02, 127.94, 128.14 (d, $J_{C\text{-}F}$ = 7.7 Hz), 128.57 (d, $J_{C\text{-}F}$ = 2.9 Hz), 132.34 (m), 132.53, 137.00 (d, $J_{C\text{-}F}$ = 2.9 Hz), 141.70 (dd, $J_{C\text{-}F}$ = 14.4 Hz, $J_{C\text{-}F}$ = 244.4 Hz), 147.37, 147.47 - 146.7 (m), 158.66, 162.02 (d, $J_{C\text{-}F}$ = 244.4)
^{19}F NMR (376.4 MHz, $CDCl_3$):	-158.82 (1F, dd, $J_{H\text{-}F}$ = 7.2 Hz, $J_{H\text{-}F}$ = 18.8 Hz, $J_{C\text{-}F}$ = 246.3 Hz, Ar*F*), -141.82 (1F, dd, $J_{H\text{-}F}$ = 7.2 Hz, $J_{H\text{-}F}$ = 18.8 Hz, $J_{C\text{-}F}$ = 248.1 Hz, Ar*F*), -116.72 (1F, tt, $J_{H\text{-}F}$ = 5.8 Hz, $J_{H\text{-}F}$ = 8.7 Hz, $J_{C\text{-}F}$ = 246.3 Hz, Ar*F*)
FT-IR (v max, cm^{-1}):	688, 707, 721, 765, 802, 821, 894, 985, 1010, 1029, 1043, 1076, 1105, 1161, 1191, 1240, 1269, 1288, 1317, 1469, 1498, 1525, 1604, 1633, 2848, 2918
MS M/Z (APCI):	657.391491 (calcd. for $C_{43}H_{52}F_3O_2$: 657.391392, M + H)
Assay (HPLC):	>99%
Assay (CHN):	Calcd. 78.63% C, 7.83% H, 13.54% rest<br>Obs. 78.664% C, 7.878% H, 13.455% rest

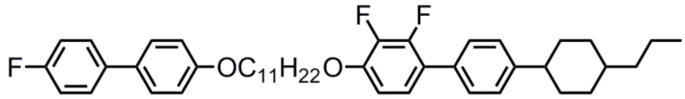

2,3-Difluoro-4-((11-((4'-fluoro-[1,1'-biphenyl]-4-yl)oxy)undecyl)oxy)-4'-(4-propylcyclohexyl)-1,1'-biphenyl (2)

Quantities used:	***i-2*** (500 mg, 1.7064 mmol), ***i-5*** (560 mg, 1.7604 mmol), potassium carbonate (470 mg, 3.41 mmol), potassium iodide (250 mg), acetone (60 ml). The general experimental procedure afforded the title compound as fine white crystals.
Yield:	0.62 g (54%)
^{1}H NMR (400 MHz, $CDCl_3$):	0.98 (3H, t, $J_{H\text{-}H}$ = 7.0 Hz, $-CH_2-C\underline{H}_3$), 1.06 (2H, m), 1.19 – 1.60 (20H, m), 1.76 – 1.96 (8H, m), 2.49 (1H, $J_{H\text{-}H}$ = 2.5 Hz, $J_{H\text{-}H}$ = 11.5 Hz, Cy$\underline{H}$), 3.97 (2H, t, $J_{H\text{-}H}$ = 6.4 Hz, $C\underline{H}_2$OAr), 4.05 (2H, t, $J_{H\text{-}H}$ = 6.4 Hz, $C\underline{H}_2$OAr), 6.76 (1H, t, $J_{H\text{-}F}$ = 7.6, Ar$\underline{H}$), 6.94 (2H, d, $J_{H\text{-}H}$ = 8.5, Ar$\underline{H}$), 7.02 – 7.12 (3H, m, Ar$\underline{H}$), 7.26 (2H, d, $J_{H\text{-}H}$ = 8.2, Ar$\underline{H}$, 7.37 – 7.49 (6H, m, Ar$\underline{H}$)
^{13}C{^{1}H} NMR (100.5 MHz, $CDCl_3$):	14.40, 20.01, 25.85, 26.02, 29.12, 29.25, 29.28, 29.35, 29.45, 29.47, 29.50, 33.52, 34.26, 36.98, 36.69, 44.31, 68.06, 69.81, 109.44, 114.78, 115.46 (d, $J_{C\text{-}F}$ = 20.7 Hz), 122.95 (d, $J_{C\text{-}F}$ =10.9 Hz), 123.42 (t, $J_{C\text{-}F}$ = 3.8 Hz), 127.47 (d, $J_{C\text{-}F}$ = 96.1 Hz), 128.13 (d, $J_{C\text{-}F}$ = 7.7 Hz), 128.56 (d, $J_{C\text{-}F}$ =3.1), 132.33, 132. 53, 136.99 (d, $J_{C\text{-}F}$ = 3.1 Hz), 141.84 (dd, $J_{C\text{-}F}$ = 15.3 Hz, $J_{C\text{-}F}$ = 246.3 Hz), 147.37, 147.51 (d, $J_{C\text{-}F}$ =2.3 Hz), 147.62 (t, $J_{C\text{-}F}$ = 2.3 Hz), 150.06 (d, $J_{C\text{-}F}$ = 11.5 Hz), 158.65, 160.79, 163.23
^{19}F NMR (376.4 MHz, $CDCl_3$):	-158.80 (1F, dd, $J_{H\text{-}F}$ = 6.9 Hz, $J_{C\text{-}F}$ = 19.6 Hz, $J_{C\text{-}F}$ = 244.7 Hz, Ar$\underline{F}$), -141.79 (1F, dd, $J_{H\text{-}F}$ = 7.6 Hz, $J_{C\text{-}F}$ = 19.6 Hz, $J_{C\text{-}F}$ = 249.7 Hz, Ar$\underline{F}$), - 116.76 - -116-.54 (1F, m, Ar$\underline{F}$)
FT-IR (ν max, cm^{-1}):	532, 594, 632, 725, 802, 894, 964, 1018, 1080, 1219, 1296, 1411, 1465, 1496, 1604, 1627, 2846, 2916
MS M/Z (LIFDI):	670.37 (calcd. for $C_{44}H_{53}F_3O_2$: 670.39977)
Assay (HPLC):	>99%

Assay (CHN):	Calcd.	78.77% C,	7.96% H,	13.27% rest
	Obs.	78.914% C,	8.029% H,	13.055% rest

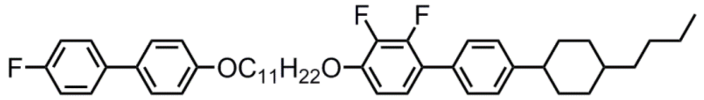

2,3-Difluoro-4-((11-((4'-fluoro-[1,1'-biphenyl]-4-yl)oxy)undecyl)oxy)-4'-(4-butylcyclohexyl)-1,1'-biphenyl (3)

Quantities used:	***i-3*** (997 mg, 2.90 mmol), ***i-5*** (1 g, 2.79 mmol), potassium carbonate (1 g, 7.30 mmol), sodium iodide (3 g), acetone (60 ml). The general experimental procedure afforded the title compound as a white solid.
Yield:	0.8 g (43%)
^{1}H NMR (400 MHz, $CDCl_3$):	0.90 (3H, t, *J* = 6.9 Hz, CH_2-CH_3), 1.06 (2H, m, Cy*H*), 1.19 – 1.60 (23H, m, Cy*H* + ArO-CH_2-(CH_2)-CH_2-OAr'), 1.73 – 1.94 (8H, m, Cy*H* + ArO-CH_2-(CH_2)-CH_2-OAr'), 2.49 (1H, tt, *J* = 3.2 Hz, *J* = 12.4 Hz, Cy*H*), 3.98 (2H, t, *J* = 6.4 Hz, ArOCH_2-CH_2), 4.05 (2H, t, *J* = 6.4 Hz, ArOCH_2-CH_2), 6.76 (1H, m, Ar*H*), 6.94 (2H, ddd, *J* = 1.8 Hz, *J* = 3.2 Hz, *J* = 8.7 Hz, Ar*H*), 7.03 – 7.12 (3H, m, Ar*H*), 7.24 – 7.29 (2H, m, Ar*H*), 7.39 – 7.51 (6H, m, Ar*H*)
^{13}C{^{1}H} NMR (100.5 MHz, $CDCl_3$):	14.16, 23.01, 25.85, 26.02, 29.14, 29.24, 29.27, 29.29, 29.36, 29.46, 29.48, 29.51, 33.57, 34.27, 37.10, 37.26, 44.32, 68.06, 69.82, 109.46 (d, J_{C-F} = 2.9 Hz), 114.78, 115.37, 115.58, 122.96 (d, J_{C-F} = 10.5 Hz), 123.44 (t, J_{C-F} = 4.8 Hz), 127.02, 127.94, 128.14 (d, J_{C-F} = 7.7 Hz), 128.57 (d, J_{C-F} = 2.9 Hz), 132.34, 132.53, 136.99 (d, J_{C-F} = 2.9 Hz), 141.70 (dd, J_{C-F} = 15.34 Hz, J_{C-F} = 247.3 Hz), 147.38, 147.44 - 146.8 (m), 158.66, 162.02 (d, J_{C-F} = 245.4)
^{19}F NMR (376.4 MHz, $CDCl_3$):	-158.81 (1F, dd, J_{H-F} = 5.8 Hz, J_{H-F} = 20.2 Hz, J_{C-F} = 246.6 Hz, Ar*F*), -141.81 (1F, dd, J_{H-F} = 5.8 Hz, J_{H-F} = 20.2 Hz, J_{C-F} = 248.3 Hz, Ar*F*), -116.71 (1F, tt, J_{H-F} = 5.8 Hz, J_{H-F} = 8.7 Hz, J_{C-F} = 245.6 Hz, Ar*F*)
FT-IR (ν max, cm^{-1}):	688, 7074, 765, 802, 821, 894, 925, 987, 1010, 1029, 1043, 1076, 1105, 1163, 1192, 1240, 1269, 1288, 1317, 1394, 1408, 1469, 1525, 1604, 1633, 2848, 2918
MS M/Z (APCI):	685.422744 (calcd. for $C_{45}H_{56}F_3O_2$: 685.422692, M + H)
Assay (HPLC):	>99%
Assay (CHN):	Calcd. 78.91% C, 8.09% H, 13.00% rest<br>Obs. 78.923% C, 8.124% H, 12.950% rest

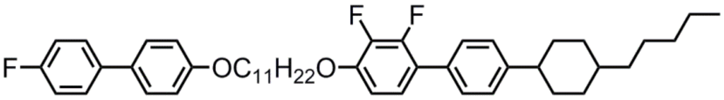

2,3-Difluoro-4-((11-((4'-fluoro-[1,1'-biphenyl]-4-yl)oxy)undecyl)oxy)-4'-(4-pentylcyclohexyl)-1,1'-biphenyl (4)

Quantities used:	***i-4*** (1038 mg, 2.90 mmol), ***i-5*** (1 g, 2.79 mmol), potassium carbonate (1 g, 7.30 mmol), sodium iodide (3 g), acetone (60 ml). The experimental procedure was as described for compound **1**, affording the title compound as a white solid.
Yield:	1.1 g (56%)
^{1}H NMR (400 MHz, $CDCl_3$):	0.88 (3H, t, J = 6.9 Hz, CH_2-CH_3), 0.07 (2H, m, CyH), 1.15 – 1.55 (25H, m, CyH + ArO-CH_2-(CH_2)-CH_2-OAr'), 1.75 – 1.95 (8H, m, CyH + ArO-CH_2-(CH_2)-CH_2-OAr'), 2.48 (1H, tt, J = 3.2 Hz, J = 11.9 Hz, CyH), 3.97 (2H, t, J = 6.9 Hz, ArOCH_2-CH_2), 4.05 (2H, t, J = 6.9 Hz, ArOCH_2-CH_2), 6.76 (1H, m, ArH), 6.94 (2H, ddd, J = 2.2 Hz, J = 2.8 Hz, J = 8.7 Hz, ArH), 7.03 – 7.11 (3H, m, ArH), 7.24 – 7.29 (2H, m, ArH), 7.38 – 7.50 (6H, m, ArH)
^{13}C{^{1}H} NMR (100.5 MHz, $CDCl_3$):	14.12, 22.72, 25.85, 26.02, 26.64, 29.14, 29.26, 29.29, 29.36, 29.46, 29.48, 29.51, 32.20, 33.57, 34.27, 37.28, 37.37, 44.33, 68.07, 69.83, 109.46 (d, J_{C-F} = 1.9 Hz), 114.76, 115.37, 115.58, 122.97 (d, J_{C-F} = 11.5 Hz), 123.46 (t, J_{C-F} = 3.8 Hz), 127.49 (d, J_{C-F} = 93.0 Hz), 128.15 (d, J_{C-F} = 7.7 Hz), 128.57 (d, J_{C-F} = 2.9 Hz), 132.34, 132.54, 137.00 (d, J_{C-F} = 2.9 Hz), 147.39, 147.48 – 147.70 (m), 158.66, 160.80, 163.25
^{19}F NMR (376.4 MHz, $CDCl_3$):	-158.83 (1F, dd, J_{H-F} = 7.2 Hz, J_{H-F} = 20.2 Hz, J_{C-F} = 244.4 Hz, ArF), -141.81 (1F, dd, J_{H-F} = 7.2 Hz, J_{H-F} = 20.2 Hz, J_{C-F} = 248.3 Hz, ArF), -116.73 (1F, tt, J_{H-F} = 4.3 Hz, J_{H-F} = 8.7 Hz, J_{C-F} = 245.1 Hz, ArF)
FT-IR (ν max, cm^{-1}):	688, 707, 802, 821, 894, 925, 987, 1010, 1029, 1043, 1076, 1107, 1122, 1163, 1192, 1240, 1269, 1288, 1319, 1394, 1408, 1469, 1498, 1525, 1604, 1635, 2848, 2918
MS M/Z (APCI):	699.438427 (calcd. for $C_{46}H_{58}F_3O_2$: 699.438342, M + H)
Assay (HPLC):	>99%
Assay (CHN):	Calcd. 79.05% C, 8.22% H, 12.73% rest
	Obs. 78.870% C, 8.266% H, 12.865% rest

1.4. Transitional Properties & Molecular Structures:

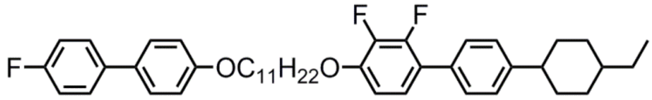

	Cr	'X' - SmA	SmA - N	N- Iso
**Transitions (°C)**	76.1	57.1	67.0	148.3
**ΔH (kJ mol^{-1})**	44.2	0.45	0.32	1.90
**ΔS/R**	15.22	0.16	0.11	0.54

Table 1: Molecular structure and transitional properties of compound **1** (RM1728)

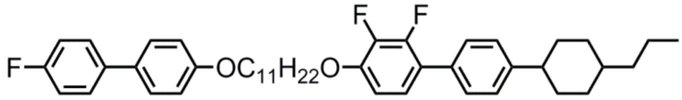

	Cr	'X' - SmA	SmA - N_{TB}	N_{TB} - N	N- Iso
**Transitions (°C)**	79.8	58.5	66.7	68.8	162.9
**ΔH (kJ mol^{-1})**	47.42	0.07	0.06	0.03	2.41
**ΔS/R**	16.16	0.03	0.02	0.01	0.67

Table 2: Molecular structure and transitional properties of compound **2** (RM1722, aka RM1081)

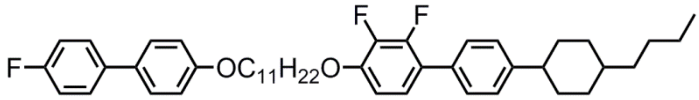

	Cr	'X' - SmA	SmA - N	N- Iso
**Transitions (°C)**	74.1	73.6	91.9	159.6
**ΔH (kJ mol^{-1})**	42.3	0.53	0.39	1.79
**ΔS/R**	14.64	0.19	0.13	0.49

Table 3: Molecular structure and transitional properties of compound **3** (RM1723)

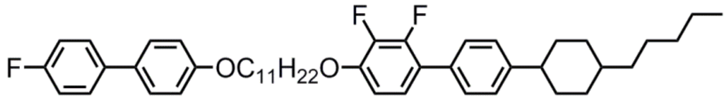

	Cr	'X' - SmA	SmA - N	N- Iso
**Transitions (°C)**	74.2	61.5	80.4	158.6
**ΔH (kJ mol^{-+1})**	43.25	0.49	0.29	2.50
**ΔS/R**	14.94	0.18	0.10	0.70

Table 4: Molecular structure and transitional properties of compound **4** (RM1724)

1.5. Supplemental NMR Spectra

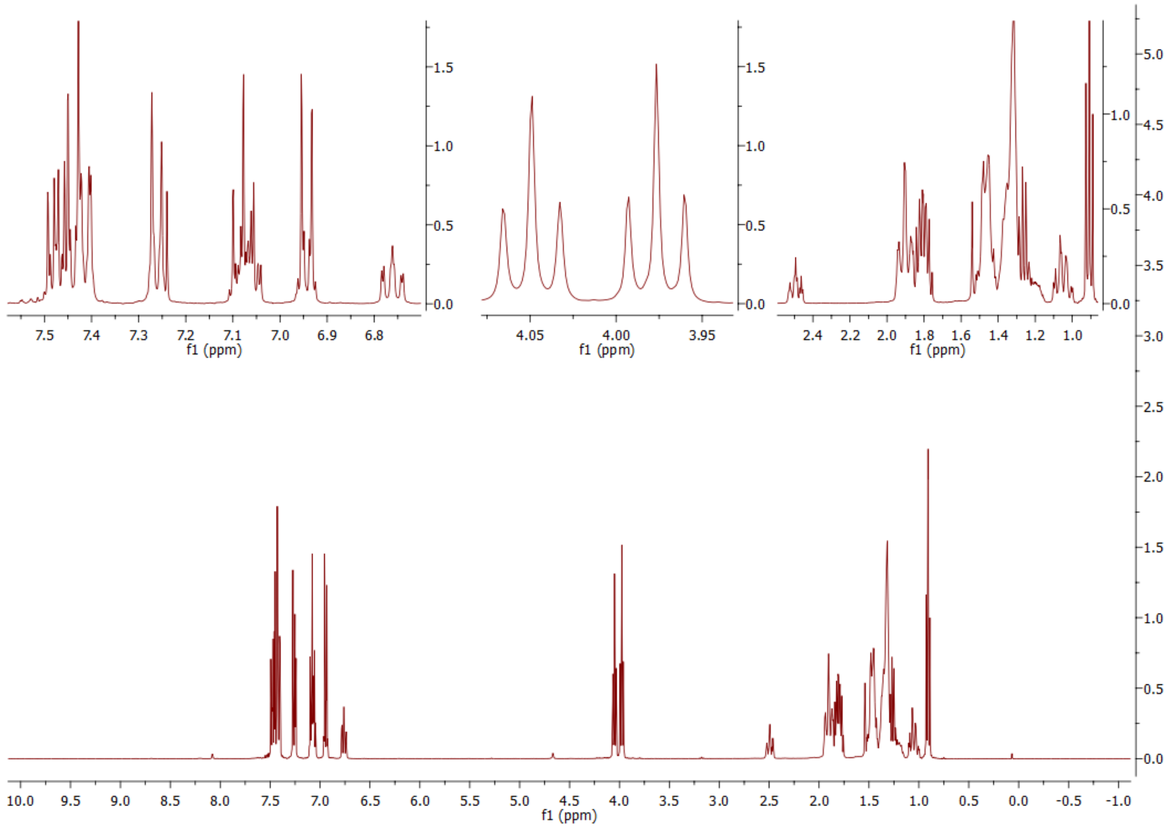

Figure SI-1: ^{1}H NMR (400 MHz, $CDCl_3$) of compound **1** with expansions.

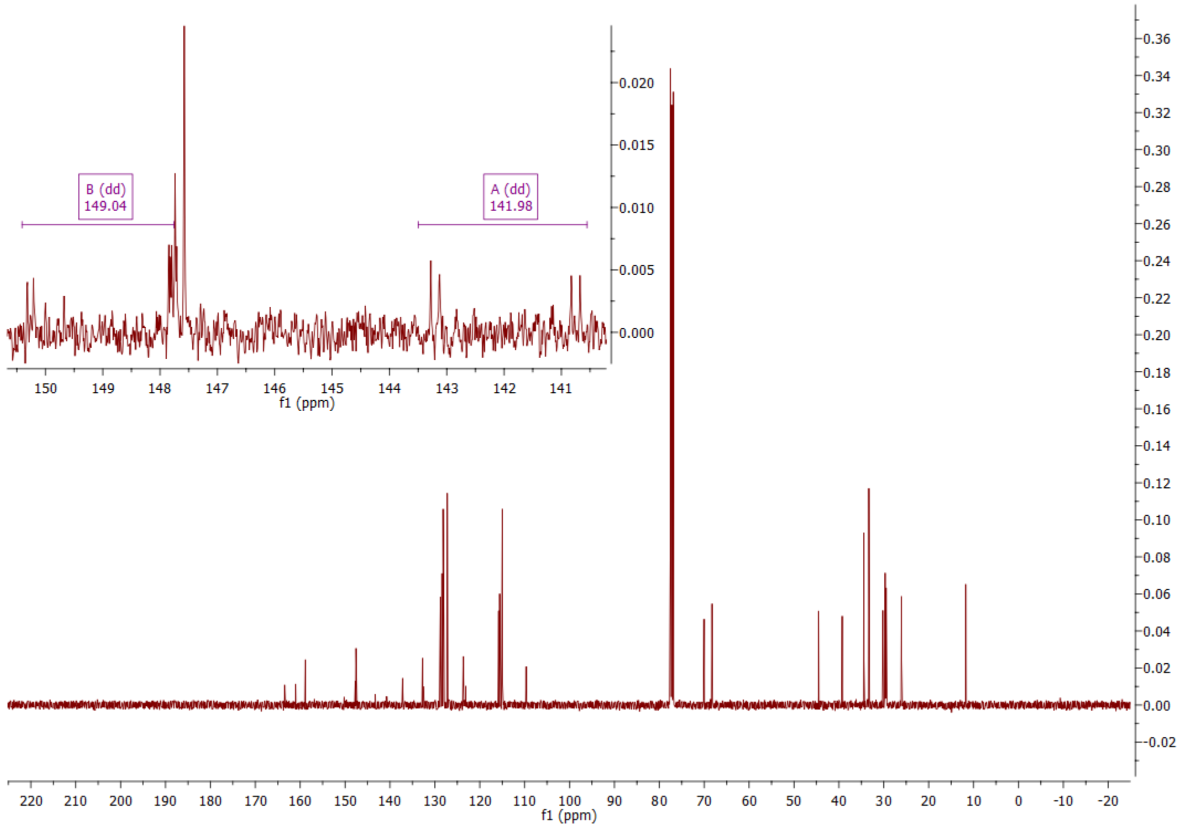

Figure SI-2: ^{13}C NMR (100.5 MHz, $CDCl_3$) of compound **1** with expansions.

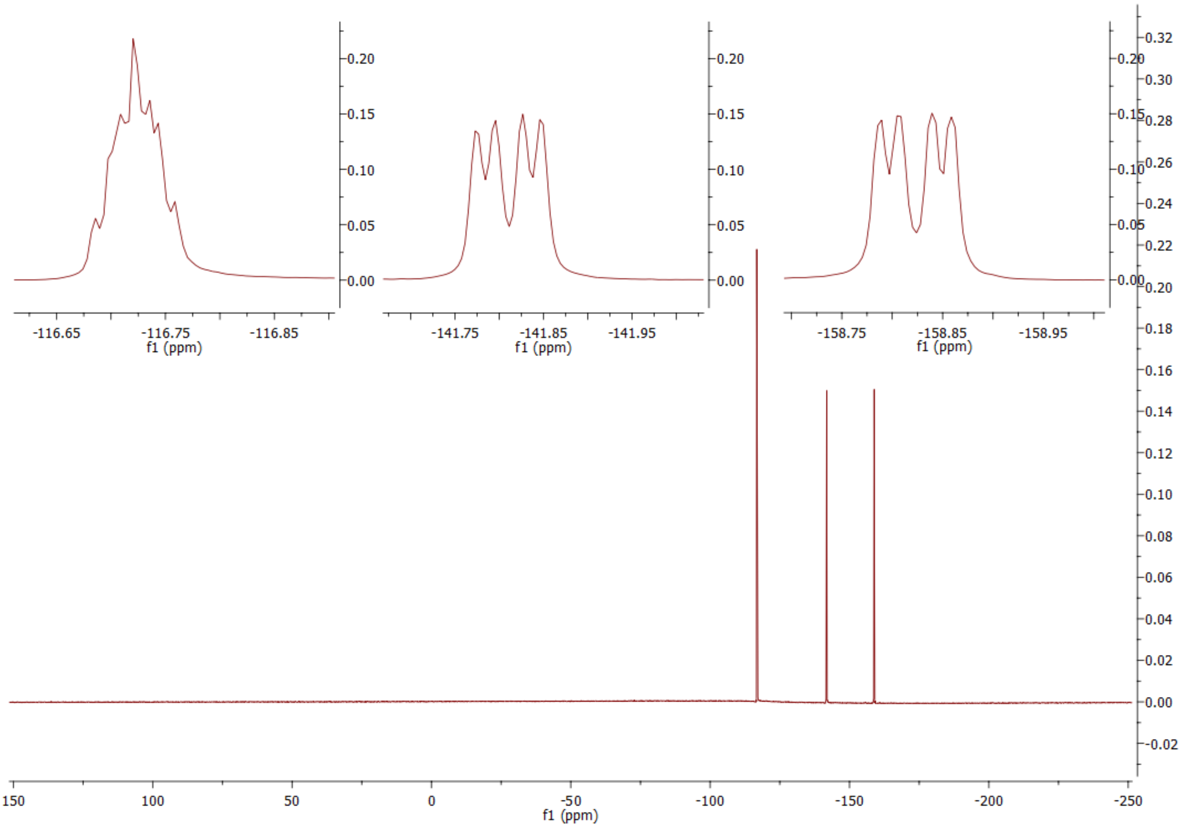

Figure SI-3: ^{19}F NMR (376.4 MHz, $CDCl_3$) of compound **1** with expansions.

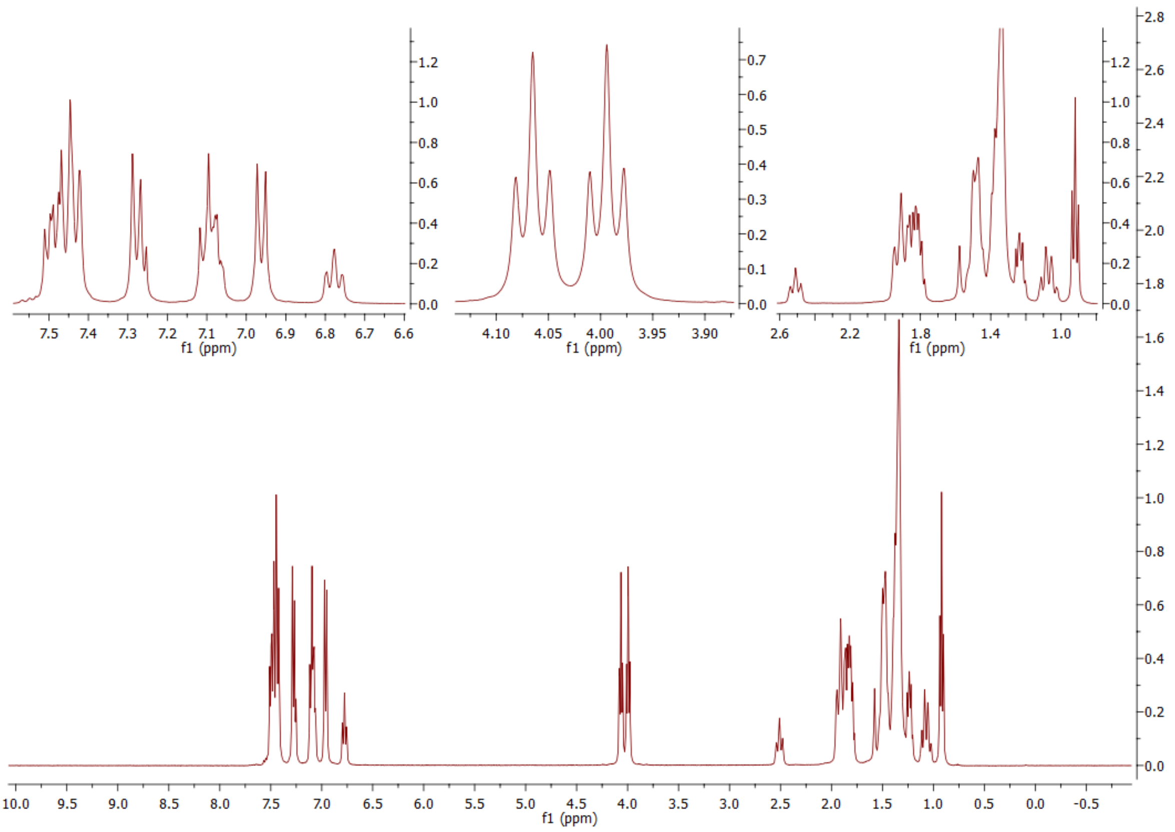

Figure SI-4: ^{1}H NMR (400 MHz, $CDCl_3$) of compound **2** with expansions.

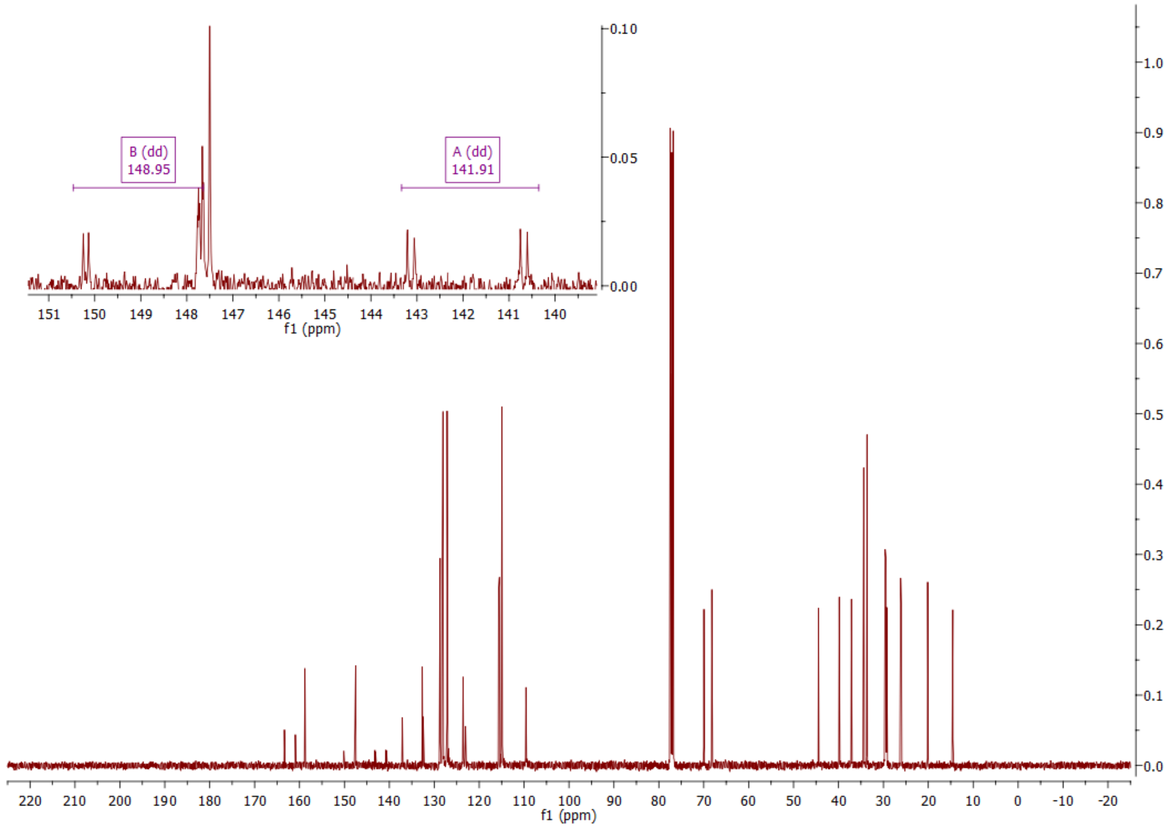

Figure SI-5: ^{13}C NMR (100.5 MHz, $CDCl_3$) of compound **2** with expansions.

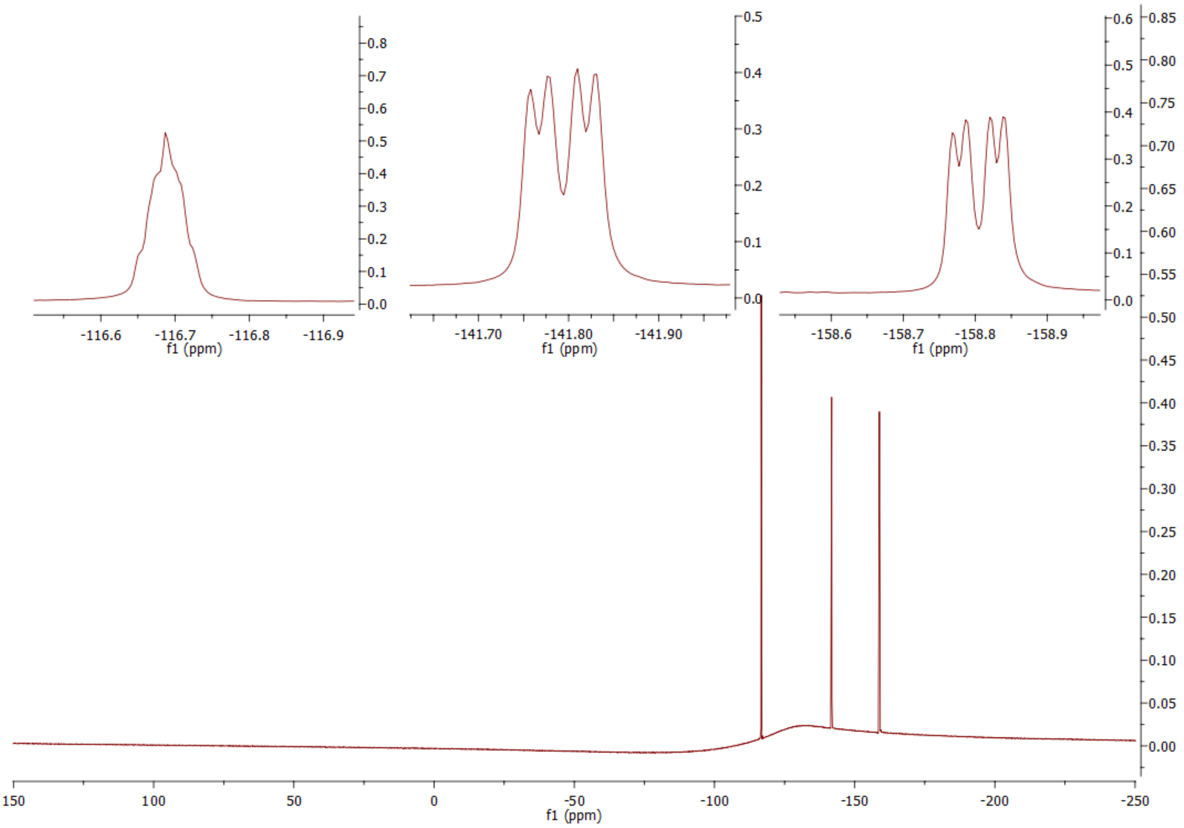

Figure SI-6: ^{19}F NMR (376.4 MHz, $CDCl_3$) of compound **2** with expansions.

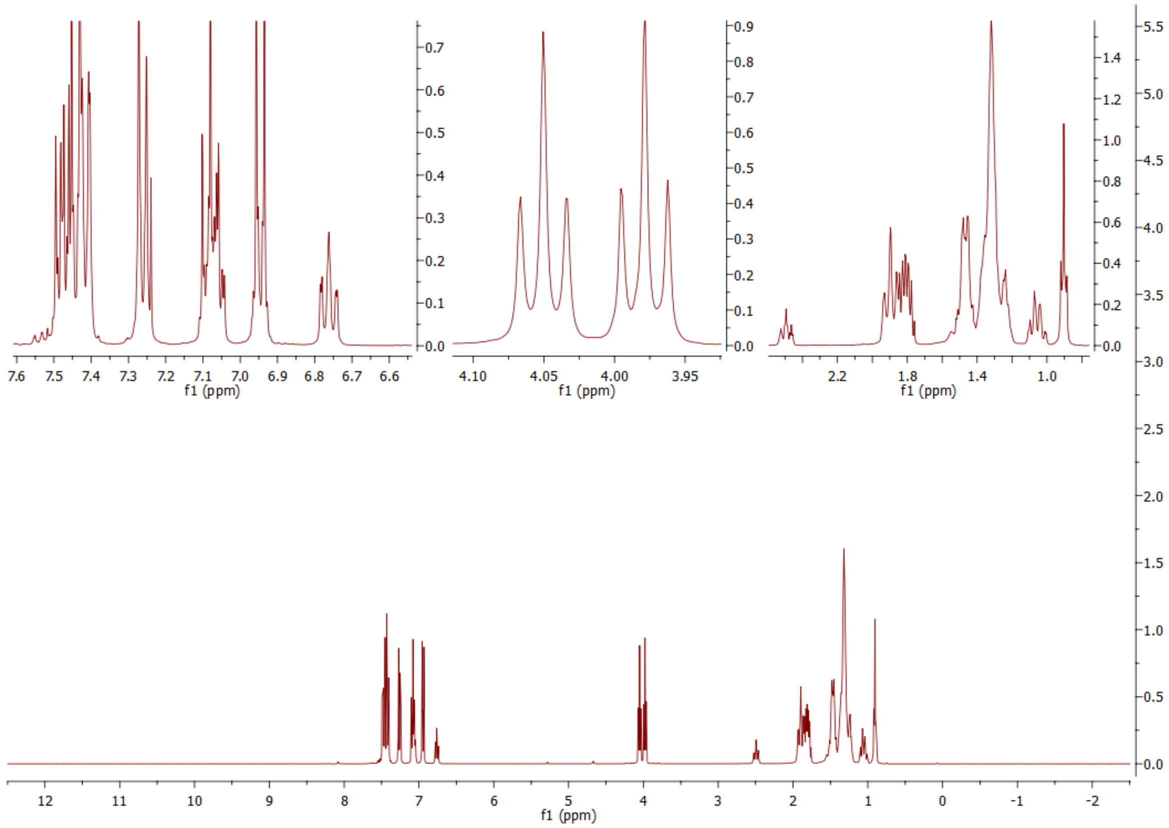

Figure SI-7: ^1H NMR (400 MHz, $CDCl_3$) of compound **3** with expansions.

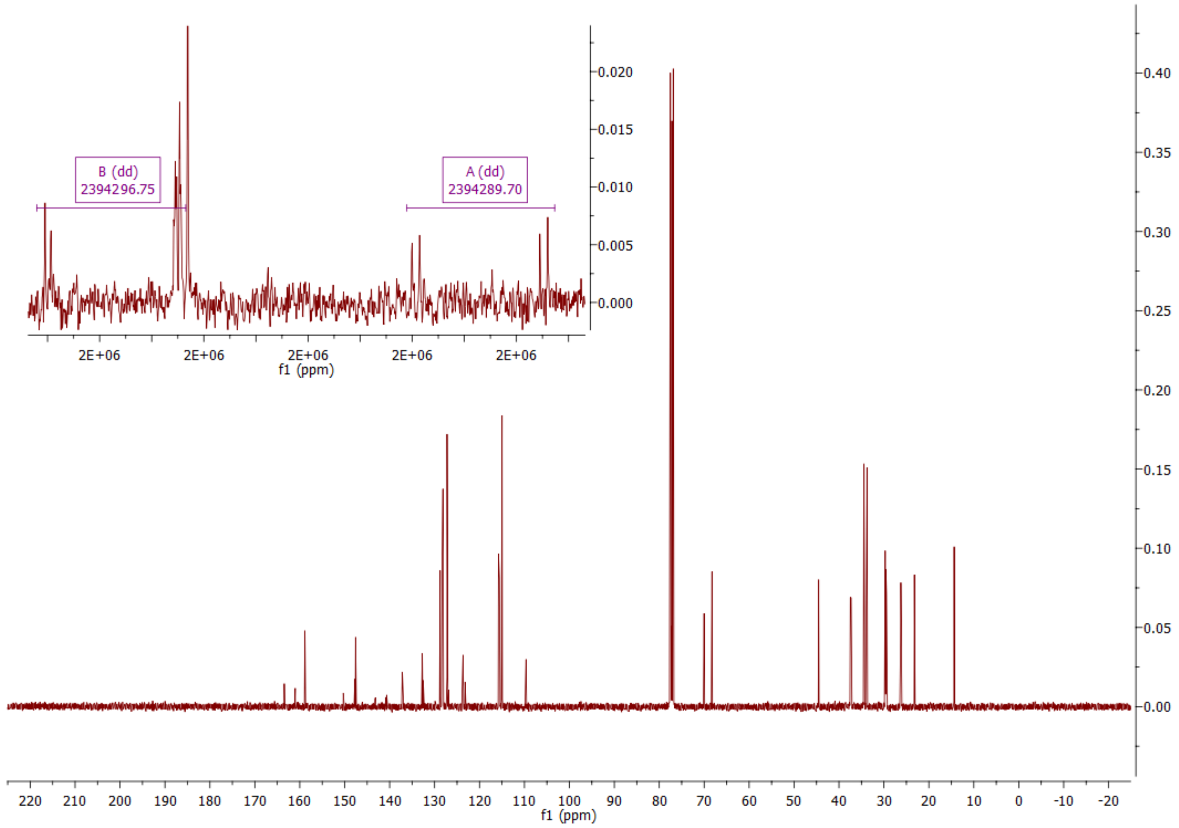

Figure SI-8: ^{13}C NMR (100.5 MHz, $CDCl_3$) of compound **3** with expansions.

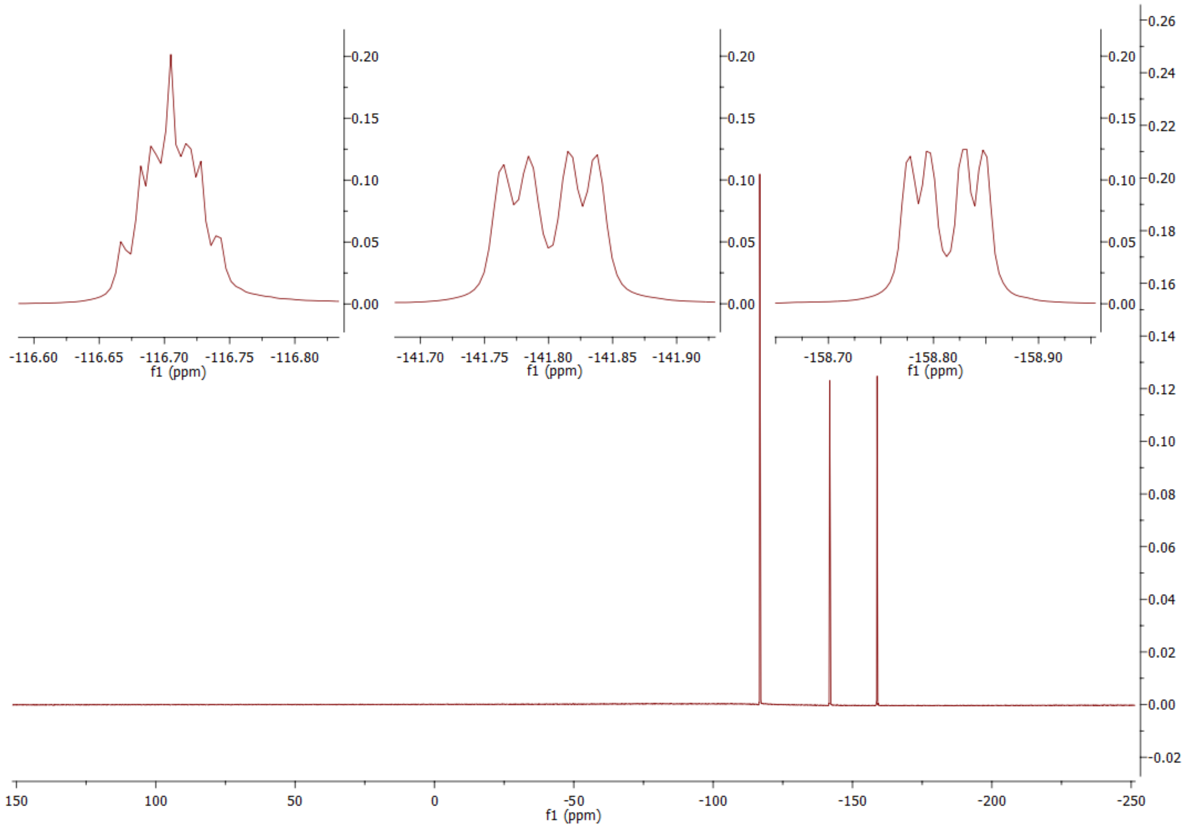

Figure SI-9: ^{19}F NMR (376.4 MHz, $CDCl_3$) of compound **3** with expansions.

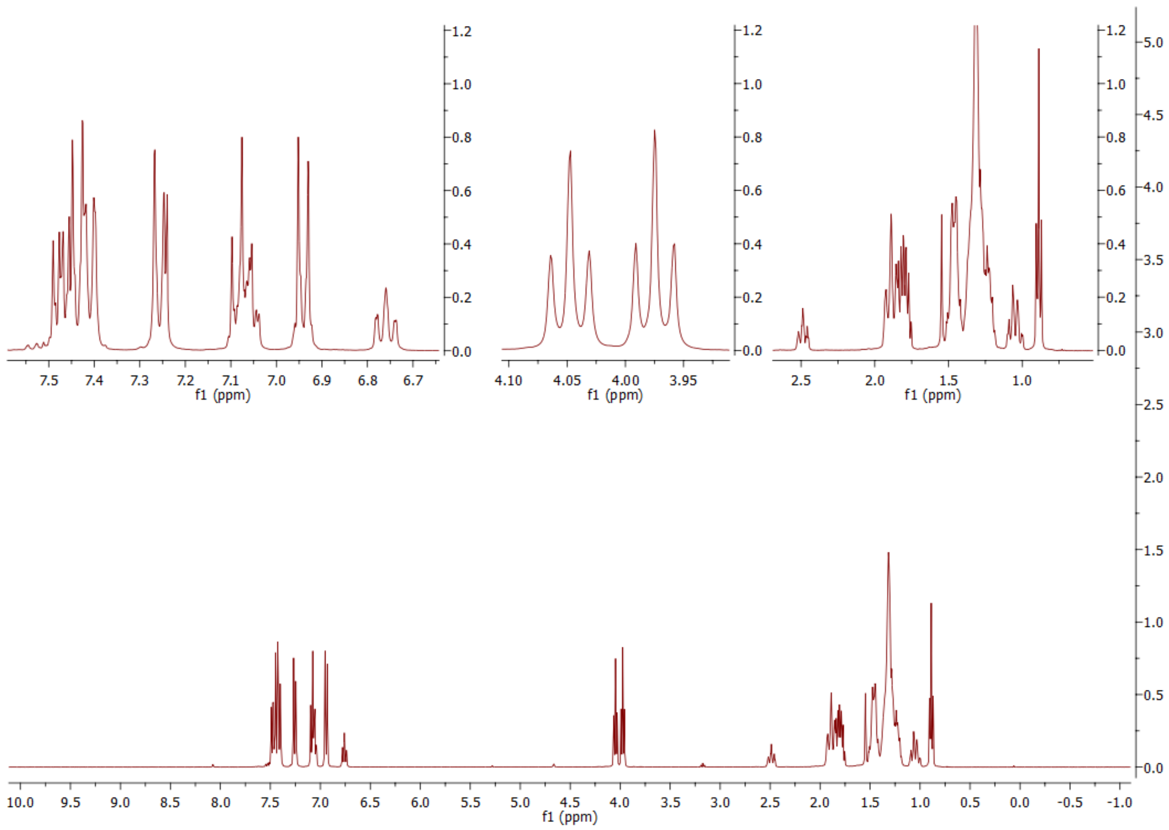

Figure SI-10: ^{1}H NMR (400 MHz, $CDCl_3$) of compound **4** with expansions.

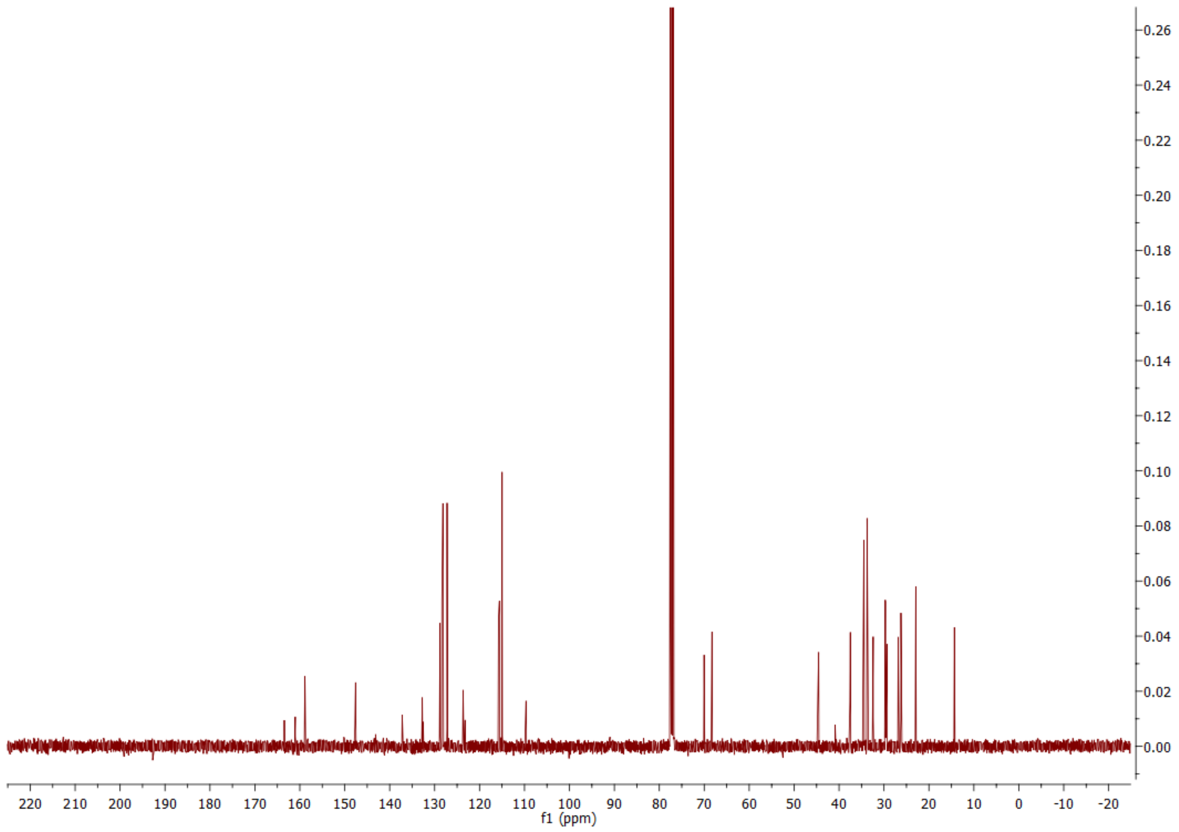

Figure SI-11: ^{13}C NMR (100.5 MHz, $CDCl_3$) of compound **4** with expansions.

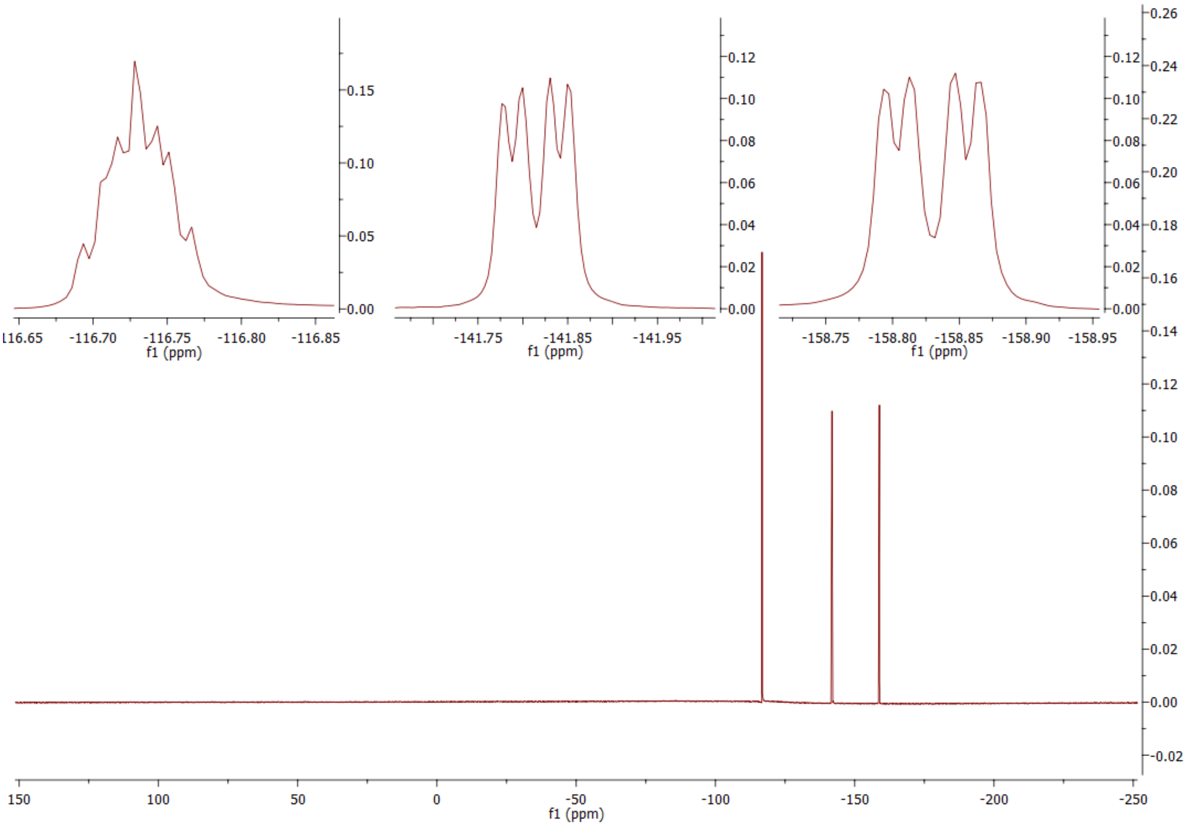

Figure SI-12: ^{19}F NMR (376.4 MHz, $CDCl_3$) of compound **4** with expansions.

1.6. Supplemental Mass Spectrometry Data

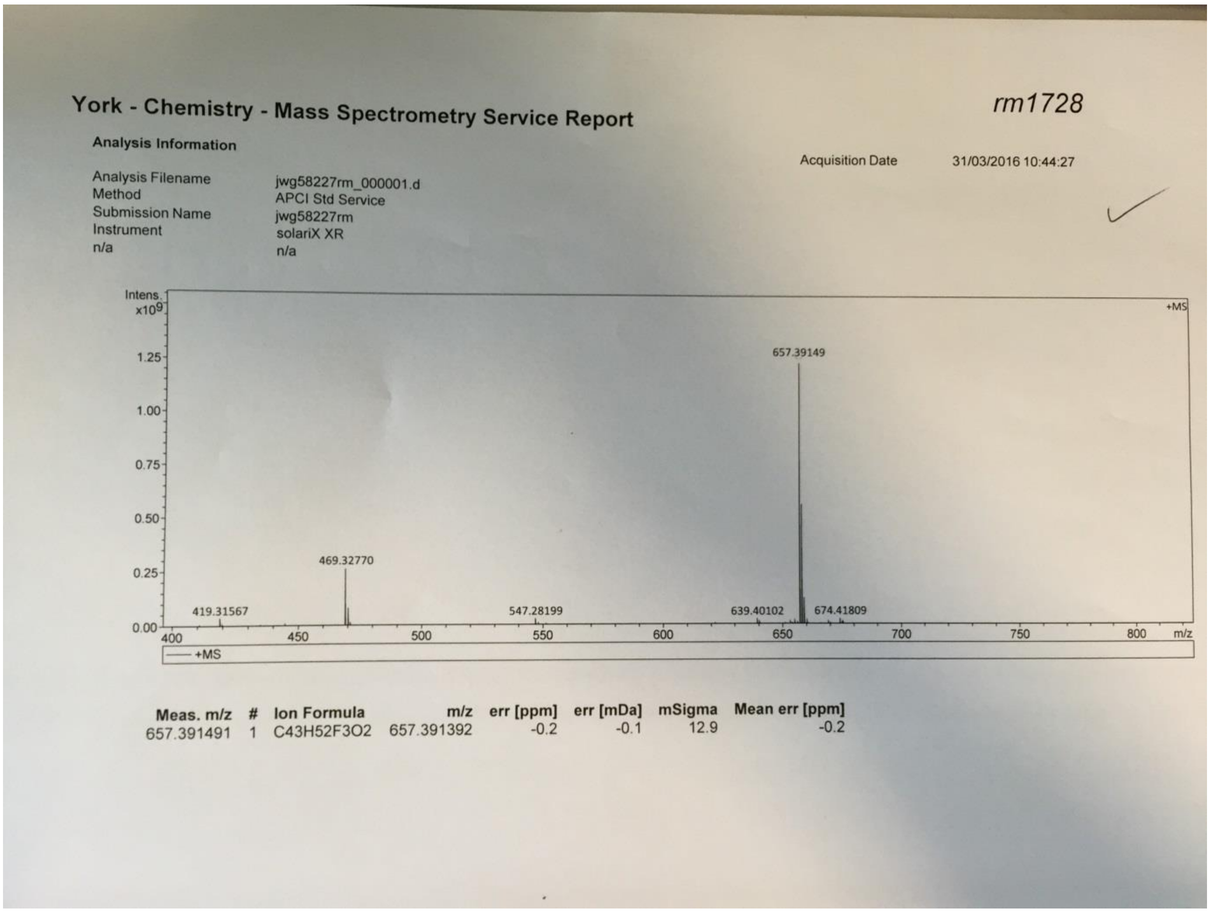
York - Chemistry - Mass Spectrometry Service Report

rm1728

Analysis Information

Acquisition Date 31/03/2016 10:44:27

Analysis Filename	jwg58227rm_000001.d
Method	APCI Std Service
Submission Name	jwg58227rm
Instrument	solariX XR
n/a	n/a

Meas. m/z	#	Ion Formula	m/z	err [ppm]	err [mDa]	mSigma	Mean err [ppm]
657.391491	1	C43H52F3O2	657.391392	-0.2	-0.1	12.9	-0.2

Figure SI-13: APCI MS data for compound **1** (RM1728)

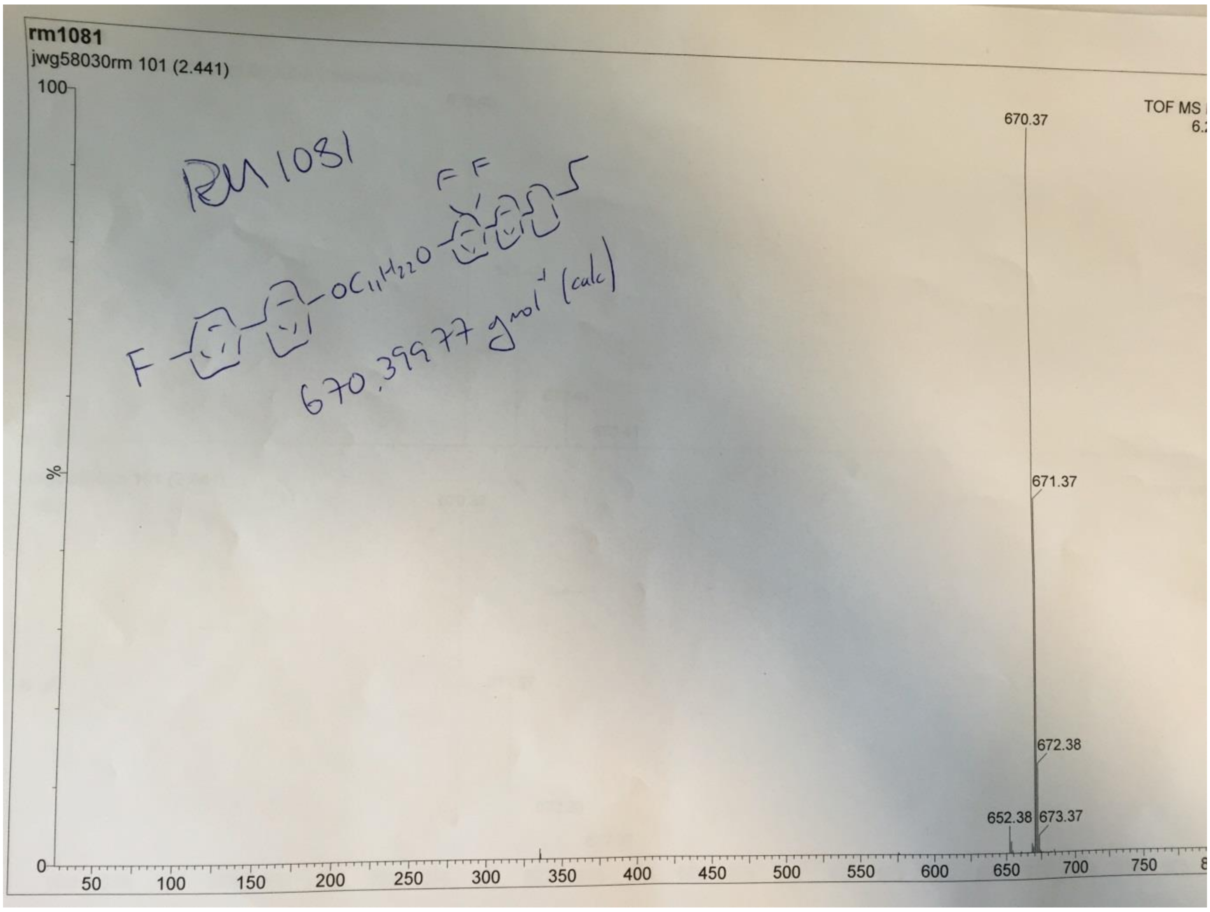

Figure SI-14: LIFDI MS data for compound **2** (RM1081)

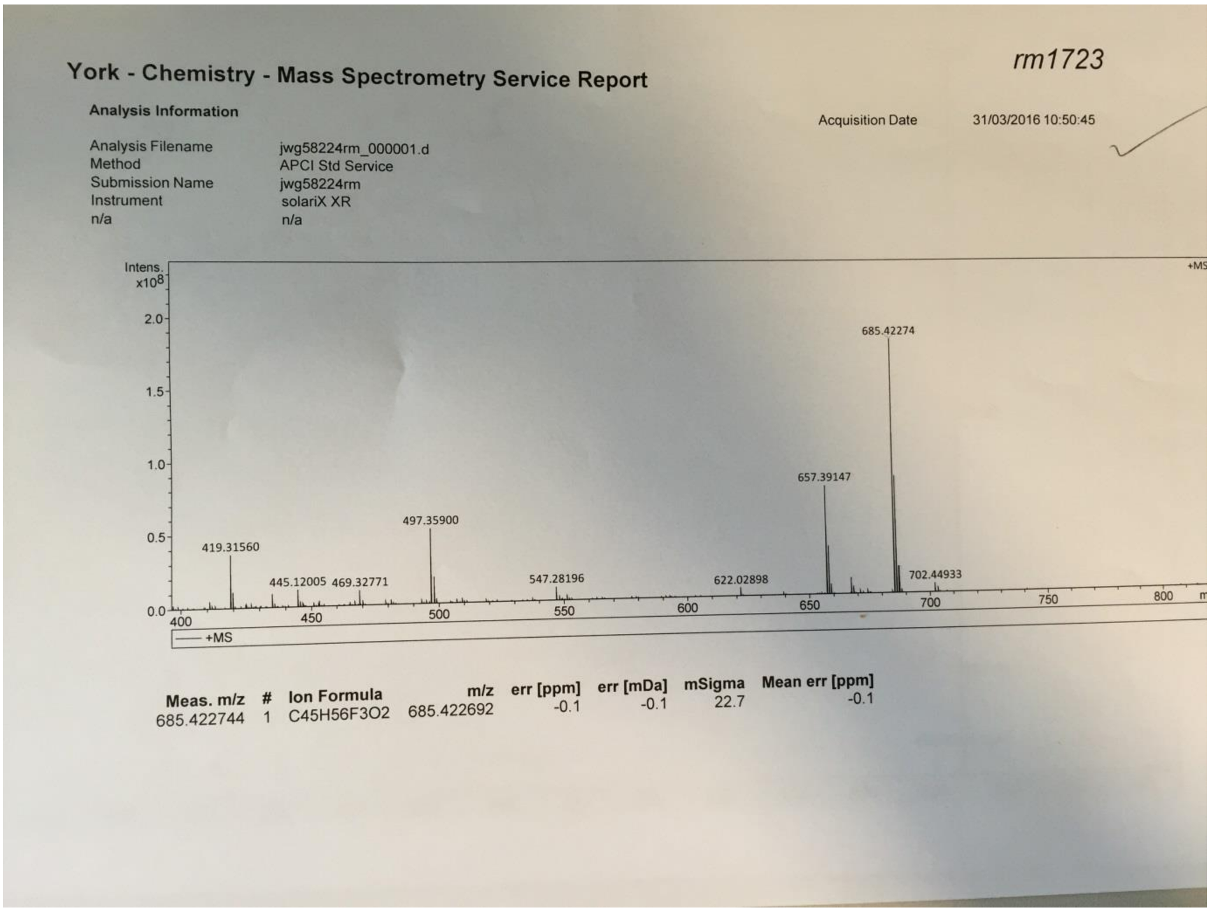

Figure SI-15: APCI MS data for compound **3** (RM1723)

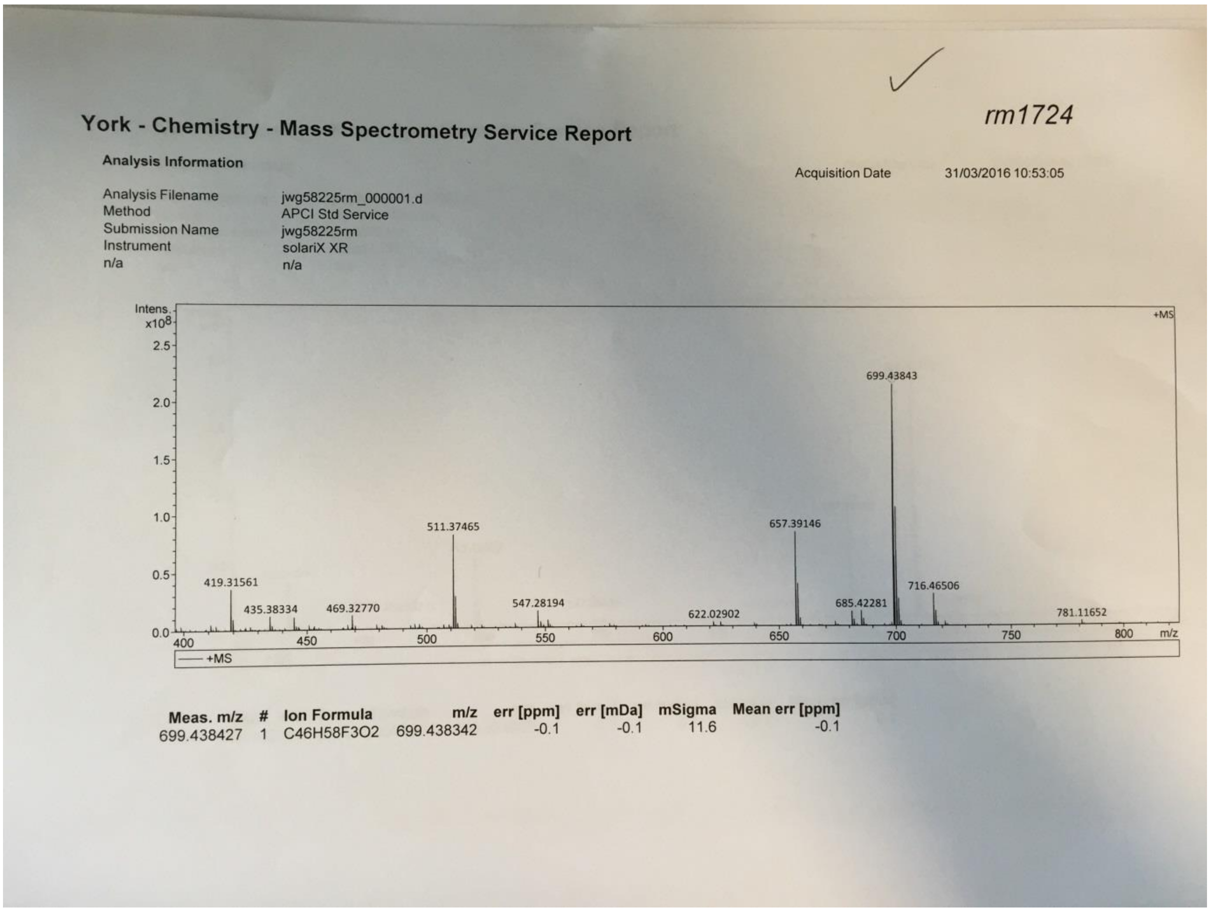

rm1724

York - Chemistry - Mass Spectrometry Service Report

Analysis Information

Acquisition Date 31/03/2016 10:53:05

Analysis Filename	jwg58225rm_000001.d
Method	APCI Std Service
Submission Name	jwg58225rm
Instrument	solariX XR
n/a	n/a

Meas. m/z	#	Ion Formula	m/z	err [ppm]	err [mDa]	mSigma	Mean err [ppm]
699.438427	1	C46H58F3O2	699.438342	-0.1	-0.1	11.6	-0.1

Figure SI-16: APCI MS data for compound **4** (RM1724)

1.7. Supplemental Microanalytical Data for 1 - 4

CHN Micro analytical Service Results

Name	Richard Mandle	Compound ID	RM1728

Element	% C	% H	% N	% Rest
Observed 1	78.67	7.90	-	13.43
Observed 2	78.66	7.86	-	13.48
Mean	78.664	7.878	-	-
Calc (theory)	78.63	7.83	-	13.54

Comments: Check std within specified limits YES / NO. Counter/run no: 22209

CHN Micro analytical Service Results

Name	Richard Mandle	Compound ID	RM1081

Element	% C	% H	% N	% Rest
Observed 1	78.95	8.04	-	13.01
Observed 2	78.88	8.02	-	13.10
Mean	78.914	8.029		-
Calc (theory)	78.77	7.96	-	13.27

Comments: Check std within specified limits YES / NO. Counter/run no: 22209

CHN Micro analytical Service Results

Name	Richard Mandle	Compound ID	RM1723

Element	% C	% H	% N	% Rest
Observed 1	78.89	8.10	-	13.01
Observed 2	78.96	8.15	-	12.89
Mean	78.923	8.124	-	-
Calc (theory)	78.91	8.09	-	13.00

Comments: Check std within specified limits YES / NO. Counter/run no: 22209

CHN Micro analytical Service Results

Name	Richard Mandle	Compound ID	RM1724

Element	% C	% H	% N	% Rest
Observed 1	78.84	8.25	-	12.91
Observed 2	78.90	8.28	-	12.82
Mean	78.870	8.266	-	-
Calc (theory)	79.05	8.22	-	12.73

Comments: Check std within specified limits YES / NO. Counter/run no: 22209

Figure SI-17: Elemental analysis data for compounds **1** (RM1728), **2** (RM1081), **3** (RM1723) & **4** (RM1724)

1.8. Supplemental Computational Chemistry Data:

All calculations pertinent to the flexibility of the central spacer were performed using the AM1 semi-empirical method as implemented in Gaussian G09. Fully relaxed scans were performed for each dihedral using 72 steps in 5 ° increments. For the sake of computational efficiency the terminal chain appended to the cyclohexyl ring was truncated to a methyl group.

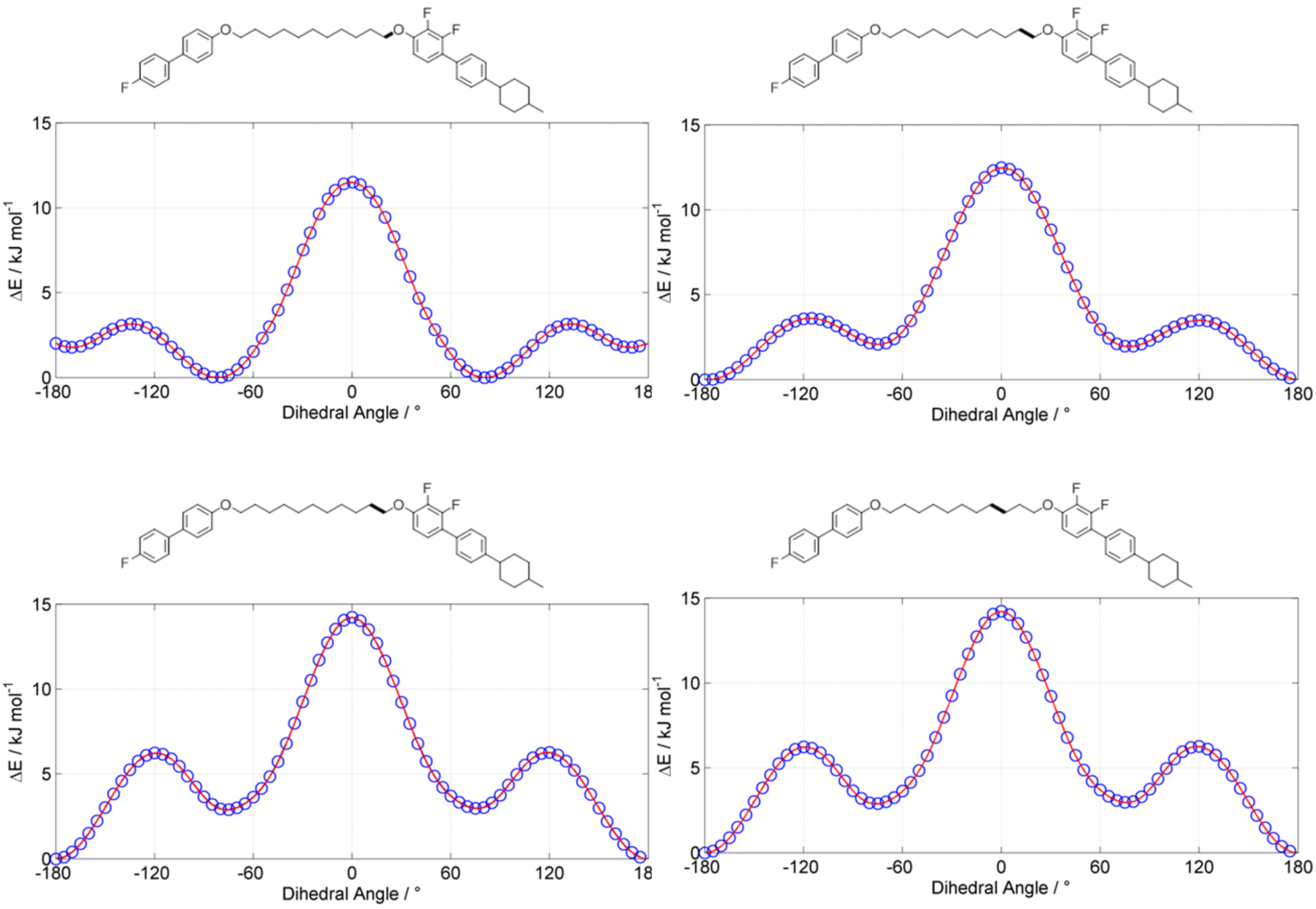

Figure SI-18: Plot of energy (kJ mol^{-1}) as a function of the dihedral angle (°) for torsions 1-4 as obtained using the AM1 semi empirical method. studied. Circles correspond to data points, the solid red line is a fit to the data to guide the eye. The *trans*/*gauche* energy difference is –1.7 kJ mol^{-1} for 1 (*gauche* is lower than *trans*), for 2 it is 2.1 kJ mol^{-1}, for 3 and 4 it is 3 kJ mol^{-1}

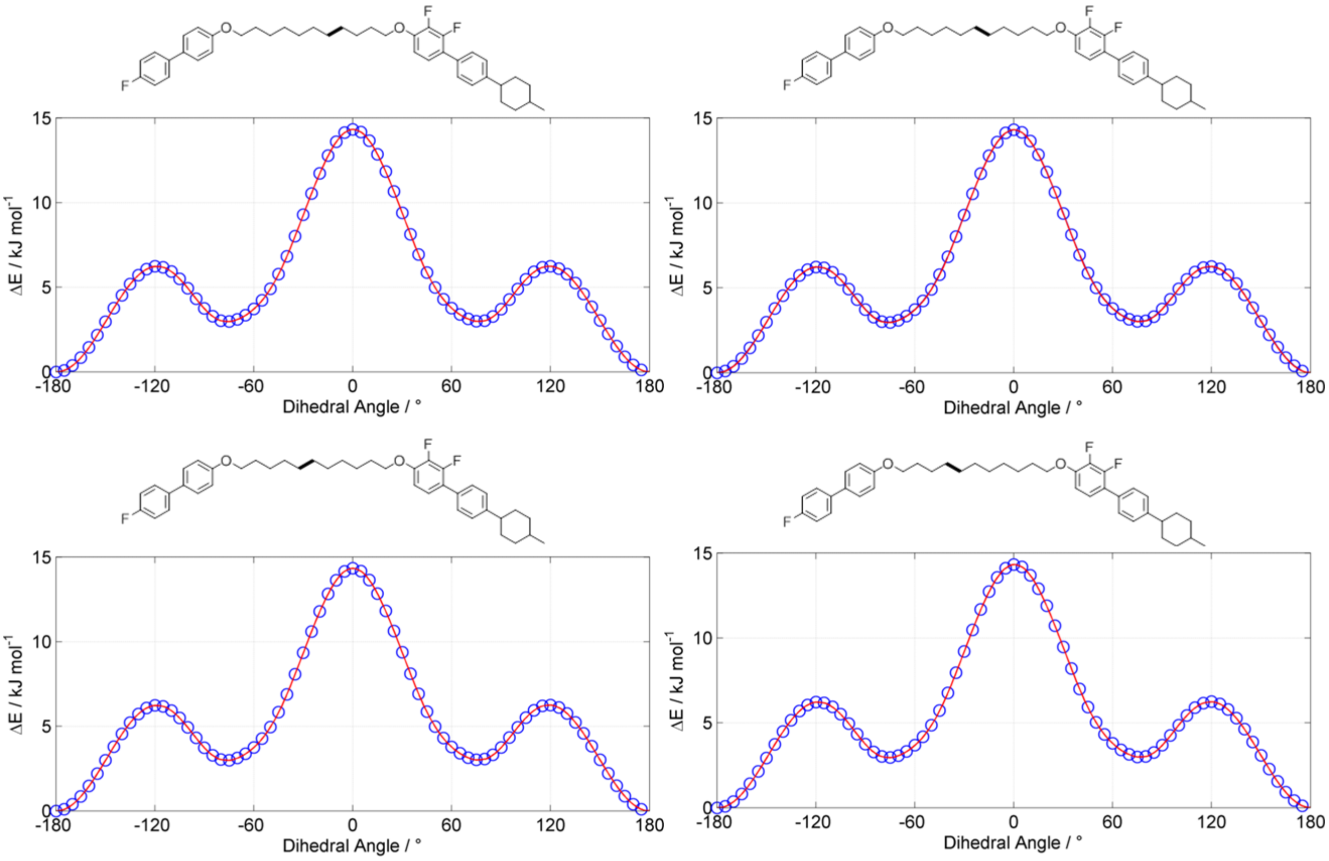

Figure SI-19: Plot of energy (kJ mol^{-1}) as a function of the dihedral angle (°) for torsions 5-8 as obtained using the AM1 semi empirical method. Circles correspond to data points, the solid red line is a fit to the data to guide the eye. For each individual torsion the *trans/gauche* energy difference is 3 kJ mol^{-1}.

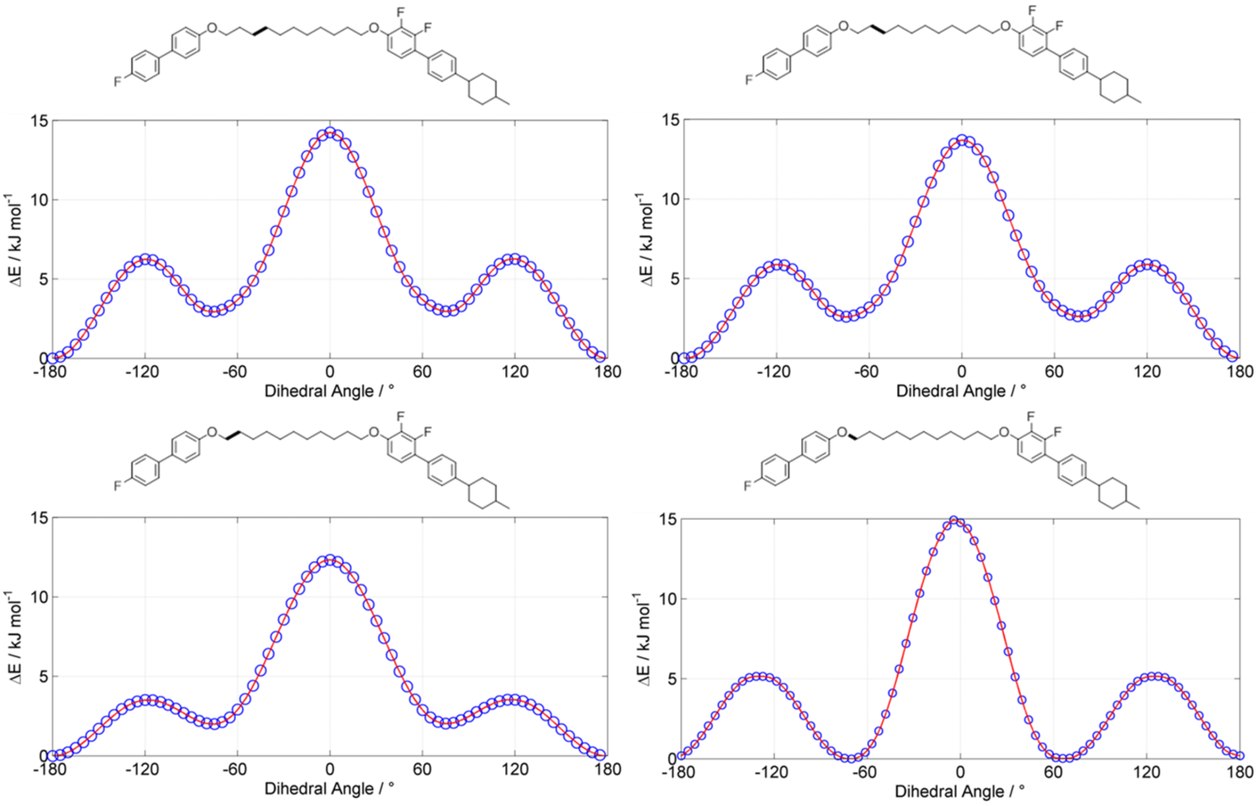

Figure SI-20: Plot of energy (kJ mol^{-1}) as a function of the dihedral angle (°) for torsions 9-12 as obtained using the AM1 semi empirical method. Circles correspond to data points, the solid red line is a fit to the data to guide the eye. The *trans*/*gauche* energy difference is 3 kJ mol^{-1} for 9, 2.6 kJ mol^{-1} for 10, 2.1 kJ mol^{-1} for 11 and -0.2 kJ mol^{-1} for 12 (*gauche* is lower in energy than *trans*).

1.9. Supplemental SAXS data

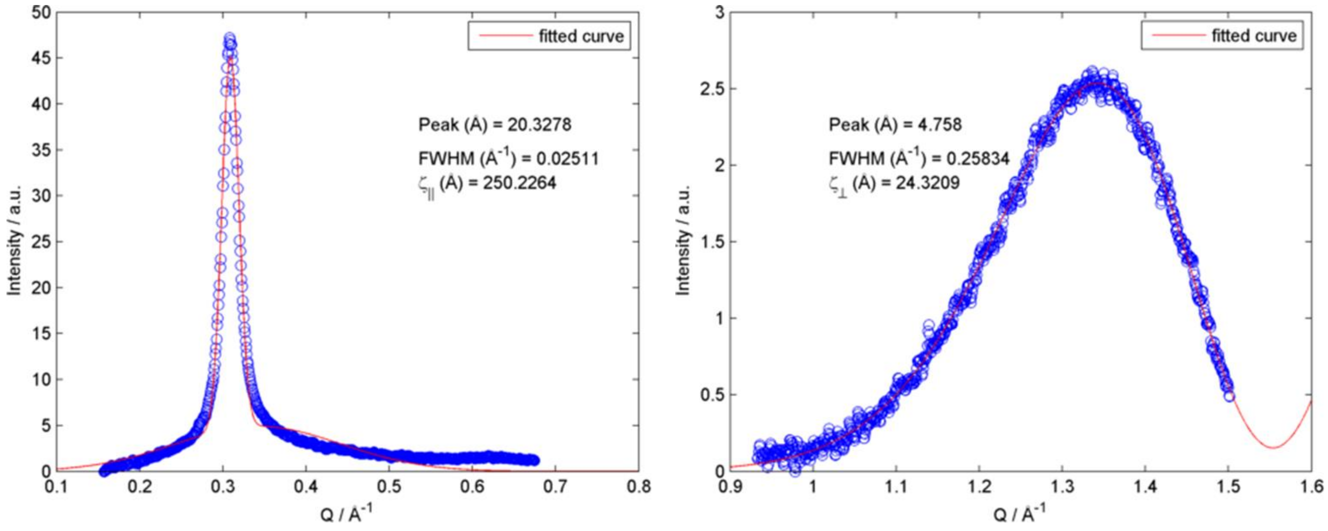

Figure SI-21: Fitting process used to extract peak positions and correlation lengths from SAXS data. Raw data (blue circles) is fitted using a 3-term Gaussian function, from the fit we determine the peak position and the full width at half maximum (FWHM), from the FWHM determination of a correlation length is trivial: $\xi = \frac{2\pi}{FWHM}$. The data and fits shown are for the small-angle (left) and wide-angle (right) scattering profiles of **3** at 92 °C, *i.e.* at the N-SmA phase transition. The R^2 values of both fits are > 0.99.

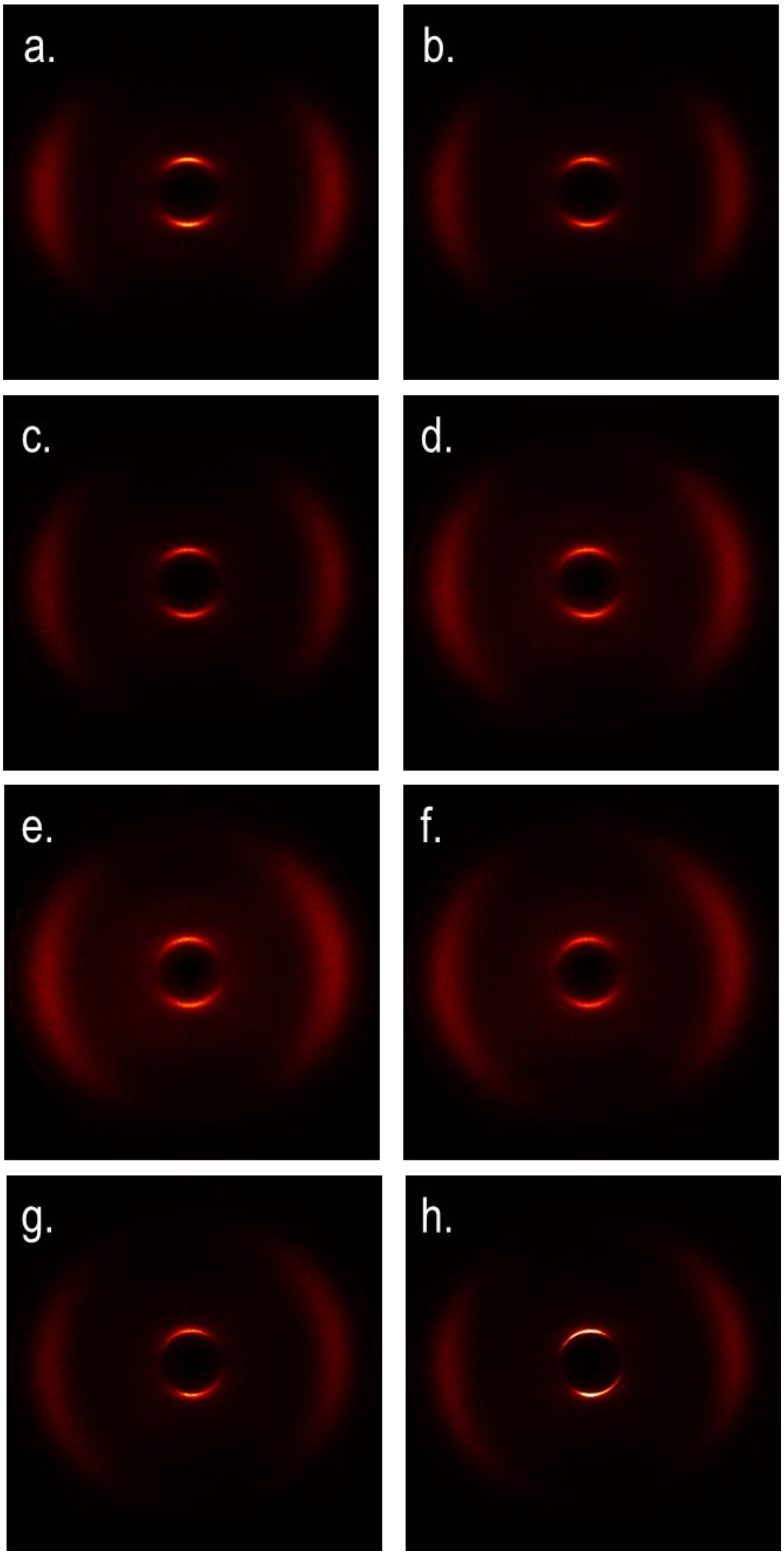

Figure SI-22: Two dimensional small angle X-ray scattering frames recorded for compound across the entire N_{TB} phase range: (a) nematic phase immediately prior to the onset of the N_{TB} phase 73 °C; (b – f) the N_{TB} phase between the temperatures of 72 and 67.7 °C, recorded in 1.5 °C intervals;

(g) at the N_{TB} – SmA transition at 66.5 °C – note the more intense scattering at small angles;

(h) the SmA phase at 65.4 °C, showing Bragg scattering at small angles.

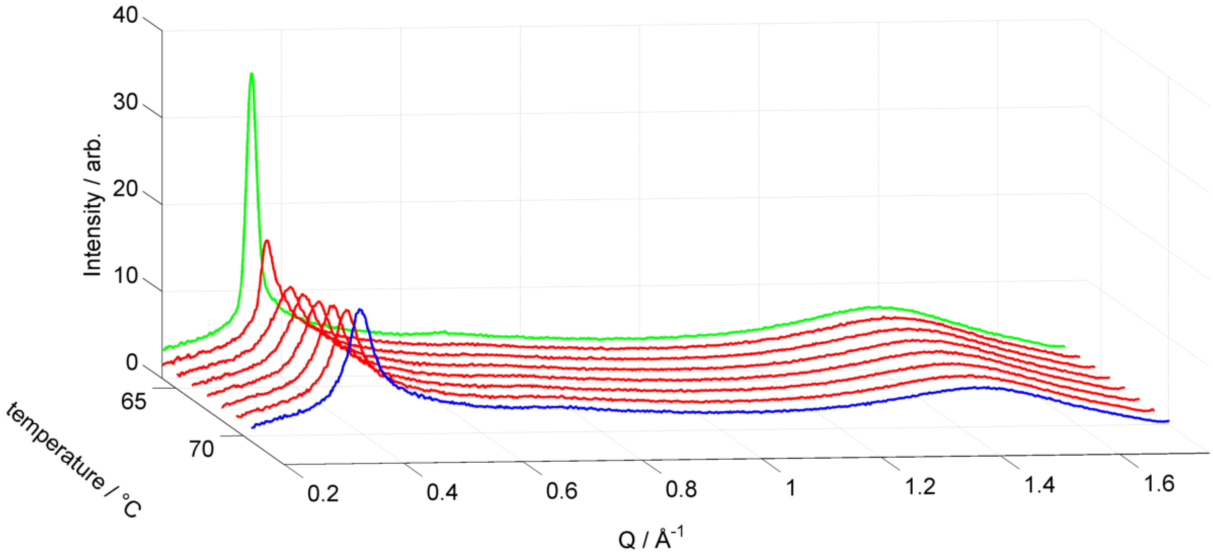

Figure SI-23: Plot of scattered X-ray intensity (arb) as a function of temperature (°C) as a function of the scattering vector, Q (Å^{-1}), for compound **2**. The blue trace is in the nematic phase at 73 °C, the red lines in the N_{TB} phase in the temperature range of 72 and 66.5 °C with ~ 1.1 °C steps, and the green line in the SmA phase at a temperature of 65.5 °C

The orientational order parameter of a liquid crystal can be determined *via* X-ray scattering as described by Davidson *et al*. 4 This method uses the intensity as a function of the azimuthal angle, I_χ, of the wide-angle scattering peak. The orientational distribution function (ODF) is expanded in terms of $\cos^{2n}$ β functions (instead of the Legendre polynomials used by Leadbetter in ref 5) as showin in equation 1:

$$f(\beta) = \sum_{n=0}^{\infty} f_{2n}\, cos^{2n}\beta \qquad (1)$$

According to Davidson the scattering profile of I_χ is related to the coefficients of the orientational distribution function by equation 2:

$$I_{\chi} = \sum_{n=0}^{\infty} f_{2n} \frac{2^n n!}{(2n+1)!!} cos^{2n}\chi \quad (2)$$

With f(b) and I_χ known it is possible to determine the order parameters $\langle P_{2n} \rangle$ *via* equation 3:

$$\langle P_{2n} \rangle = \frac{\int_0^{\pi/2} P_{2n} f(\beta)\sin(\beta)\, d\beta}{\int_0^{\pi/2} f(\beta)\sin(\beta)\, d\beta}; n = 1, 2. \qquad (3)$$

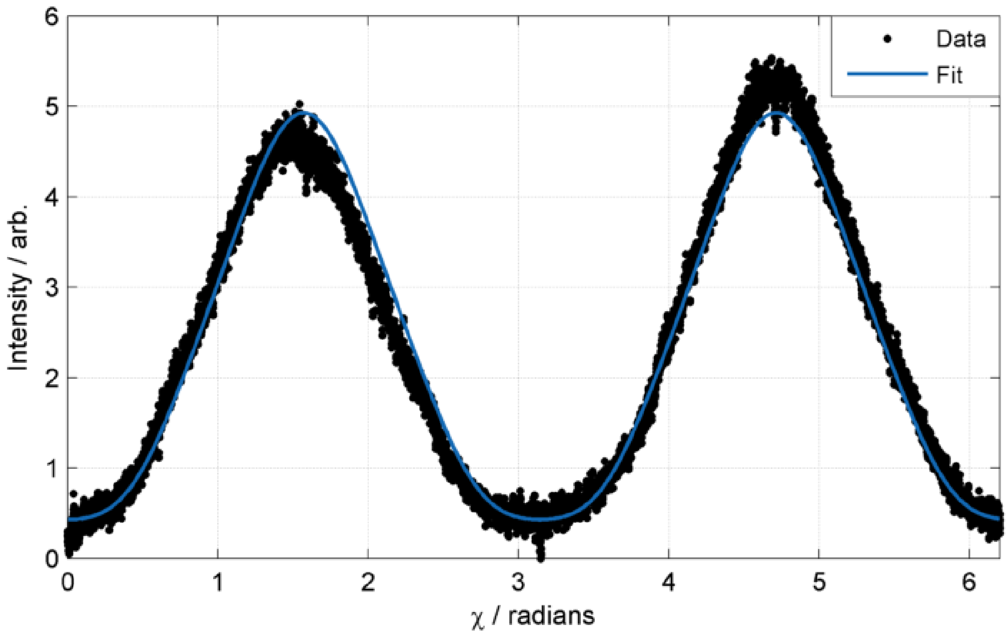

Figure SI-24: SAXS data for **2** at a reduced temperature (T - $T_{N\text{-}Iso}$) of -6, fitted as described above.

References:

1. Mandle R.J., Davis E.J., Lobato S.A., Vol C.-C.A., Cowling S.J. and Goodby J.W., “Synthesis and Characterisation of an Unsymmetrical, Ether-linked, Fluorinated Bimesogen Exhibiting a New Polymorphism Containing the NTB or ‘Twist-Bend’ Phase”, *PCCP*, **2014**, *16*, 6907 – 6915.
2. Mandle R.J., Voll C.C.A., Lewis D.J., Goodby J.W., “Etheric Bimesogens and the Twist-Bend Nematic Phase”, *Liq. Cryst.,* **2015**, *43*, 13-21.
3. Gaussian 09, Revision E.01, Frisch, M. J.; Trucks, G. W.; Schlegel, H. B.; Scuseria, G. E.; Robb, M. A.; Cheeseman, J. R.; Scalmani, G.; Barone, V.; Mennucci, B.; Petersson, G. A.; Nakatsuji, H.; Caricato, M.; Li, X.; Hratchian, H. P.; Izmaylov, A. F.; Bloino, J.; Zheng, G.; Sonnenberg, J. L.; Hada, M.; Ehara, M.; Toyota, K.; Fukuda, R.; Hasegawa, J.; Ishida, M.; Nakajima, T.; Honda, Y.; Kitao, O.; Nakai, H.; Vreven, T.; Montgomery, J. A., Jr.; Peralta, J. E.; Ogliaro, F.; Bearpark, M.; Heyd, J. J.; Brothers, E.; Kudin, K. N.; Staroverov, V. N.; Kobayashi, R.; Normand, J.; Raghavachari, K.; Rendell, A.; Burant, J. C.; Iyengar, S. S.; Tomasi, J.; Cossi, M.; Rega, N.; Millam, J. M.; Klene, M.; Knox, J. E.; Cross, J. B.; Bakken, V.; Adamo, C.; Jaramillo, J.; Gomperts, R.; Stratmann, R. E.; Yazyev, O.; Austin, A. J.; Cammi, R.; Pomelli, C.; Ochterski, J. W.; Martin, R. L.; Morokuma, K.; Zakrzewski, V. G.; Voth, G. A.; Salvador, P.; Dannenberg, J. J.; Dapprich, S.; Daniels, A. D.; Farkas, Ö.; Foresman, J. B.; Ortiz, J. V.; Cioslowski, J.; Fox, D. J. Gaussian, Inc., Wallingford CT, 2009.
4. P. Davidson, D. Petermann and A. M. Levelut, The Measurement of the Nematic Order Parameter by X-ray Scattering Reconsidered, *J. Phys. II France,* **1995**, *5*, 113-131, DOI: 10.1051/jp2:1995117
5. A. J. Leadbetter, E. K. Norris, *Mol. Phys*. **1979**, 38–3, 669.
